# Pleiotropic effects of schizophrenia-associated genetic variants in neuron firing and cardiac pacemaking revealed by computational modeling

**DOI:** 10.1038/s41398-017-0007-4

**Published:** 2017-11-17

**Authors:** Tuomo Mäki-Marttunen, Glenn T. Lines, Andrew G. Edwards, Aslak Tveito, Anders M. Dale, Gaute T. Einevoll, Ole A. Andreassen

**Affiliations:** 1 NORMENT, KG Jebsen Centre for Psychosis Research, Institute of Clinical Medicine, University of Oslo, Oslo, Norway; 2 Simula Research Laboratory and Center for Cardiological Innovation, Oslo, Norway; 3 Multimodal Imaging Laboratory, UC San Diego, La Jolla, CA USA; 4 Department of Neurosciences, University of California San Diego, La Jolla, CA USA; 5 Department of Radiology, University of California, San Diego, La Jolla, CA USA; 6 Department of Mathematical Sciences and Technology, Norwegian University of Life Sciences, Ås, Norway; 7 Department of Physics, University of Oslo, Oslo, Norway; 8 Division of Mental Health and Addiction, Oslo University Hospital, Oslo, Norway

## Abstract

Schizophrenia patients have an increased risk of cardiac dysfunction. A possible factor underlying this comorbidity are the common variants in the large set of genes that have recently been discovered in genome-wide association studies (GWASs) as risk genes of schizophrenia. Many of these genes control the cell electrogenesis and calcium homeostasis. We applied biophysically detailed models of layer V pyramidal cells and sinoatrial node cells to study the contribution of schizophrenia-associated genes on cellular excitability. By including data from functional genomics literature to simulate the effects of common variants of these genes, we showed that variants of voltage-gated Na^+^ channel or hyperpolarization-activated cation channel-encoding genes cause qualitatively similar effects on layer V pyramidal cell and sinoatrial node cell excitability. By contrast, variants of Ca^2+^ channel or transporter-encoding genes mostly have opposite effects on cellular excitability in the two cell types. We also show that the variants may crucially affect the propagation of the cardiac action potential in the sinus node. These results may help explain some of the cardiac comorbidity in schizophrenia, and may facilitate generation of effective antipsychotic medications without cardiac side-effects such as arrhythmia.

## Introduction

Schizophrenia (SCZ) is a heritable mental disorder with a high burden of morbidity and large social impacts^1^. A recent genome-wide association study (GWAS) has identified more than a hundred genetic loci exceeding genome-wide significance^2^. The loci implicate genes that encode numerous ion channel subtypes and calcium transporters and are major contributors to the functions of cells in not only brain but also organs outside the central nervous system, such as heart. Evidence for increased cardiac dysfunction in SCZ patients are shown by meta-studies that reported a 2.5–3-fold increase in mortality rates^3,4^. Approximately 40% of the excess deaths are caused by accidents and suicides, while the remaining 60% are natural^5^—and largely due to cardiovascular disease^6^. Some of these excess deaths are linked to the increased risk of sudden cardiac death conveyed by long-term use of antipsychotic drugs^7^, many of which are known to have side-effects related to arrhythmia, including prolongation of QT interval^8^ and torsades de pointes^9^. In parallel to these observations, GWASs of cardiac phenotypes, such as electrocardiographic (ECG) measures, highlight a set of genes that overlaps with the one discovered in GWASs of SCZ^10,11^. Nevertheless, both the genetic and mechanistic connections between cardiac and neural phenotypes in SCZ patients remain poorly understood. Here, we attempt to combine our recently developed genetic approaches with biophysical models of well-characterized cardiac and neuronal cell types to provide general mechanistic links between neural and cardiac tissue for SCZ-associated variants.

It is of key relevance to know if there is an inherent, genetic risk in addition to the external, drug-induced risks in the treatment of SCZ that may underlie the comorbidity between cardiac disease and SCZ—the SCZ-associated single-nucleotide polymorphisms (SNPs) might, however, as well be protective against cardiac disease. The effects of primarily brain disorder-related SNPs on cardiac phenotypes is a largely unexplored area, while there are a few examples of the opposite: SNPs that were first identified by a cardiac disease and then found to convey a risk of brain dysfunction, such as seizures^12,13^. The cellular homogeneity in the heart, in contrast to the heterogeneity in both structure and function of the brain, is an important aid in uncovering cross-tissue functional genetics in both approaches.

SCZ is associated with genes affecting transmembrane currents of all major cationic species, Na^+^, K^+^, and Ca^2+^ (ref. 2). In addition, some of the SCZ-linked genes are involved in regulation of intracellular Ca^2+^ dynamics^2^, which importantly modulate cellular excitability in both the heart and brain, via a range of Ca^2+^-sensitive plasma membrane current carriers. The rise of biophysical modeling of neurons^14^ and cardiac pacemaker cells^15^ provide a solid basis for analyzing this intrinsic excitability as a coordinated interplay of ion channels and ion transporters. In addition, there is an increasing amount of *in vitro* data on the effect of genetic variations on such ion channel or calcium transporter functions, and much of these data can be implemented in the biophysical models. This opens the door for a mechanistic analysis of SCZ-related genes^16^, mapping the functional genomics data to predictions of cellular function and dysfunction both in neural and cardiac tissue.

In this work, we use computational modeling to study the contribution of SCZ-associated genes to cardiac and neuronal excitability. We focus our analyses on two well-studied cell types that are central to cortical information processing and cardiac pacemaking, namely, layer V pyramidal cells (L5PCs) in the cortex and sinoatrial node cells (SANCs) in the myocardium. The apical tuft of an L5PC integrates non-local synaptic inputs, and is considered a biological substrate for cortical associations providing high-level context for low-level (e.g., sensory) inputs that arrive at the perisomatic compartments^17^. Therefore, the ability of L5PC to integrate the apical and perisomatic inputs has been proposed as one of the mechanisms that could be impaired in hallucinating patients^17^. The SANCs, in turn, have a key role in controlling heart rate as the primary pacemakers of the mammalian heart. While SANCs derive from cardiac lineage and are, therefore, regarded as a specialized form of myocardium, their morphology, electrophysiology, and ion-channel expression profiles are the most neuron-like of any studied cardiac muscle cell type. As such, they represent a population of cardiac cells that may be most apt to display functional alterations as a result of SCZ-associated variants. We apply two recent L5PC models^18,19^ and two recent SANC models^20,21^ to argue for the generality of our findings.

We show that subtle SNP-like variants of ion-channel and calcium-transporter-encoding genes cause notable effects in intrinsic excitability of both neurons and heart cells. Our approach is limited by the data concerning the functional effects of the SCZ-related common genetic variants. To overcome this limitation, we concentrate on a set of *in vitro*-observed effects of more extreme genetic variations, as described previously^16^. A key assumption of our approach is that the effects of SNP variants can be represented as downscaled versions of the more extreme variants, and that the emergence of disease phenotypes results from the combined effect of a large number of subtle SNP effects^22,23^. Our results contribute to explaining some of the cardiac comorbidity in schizophrenia, and could form the basis for development of antipsychotic medications that are free from cardiac side-effects.

## Materials and methods

### Models of neurons and cardiac pacemaker cells

We apply two multicompartmental L5PC models, “Hay”^18^ and “Almog”^19^ model, and two single-compartment SANC models, “Kharche”^20^ and “Severi”^21^ model. These models include Hodgkin-Huxley type description for channel activation and inactivation, and hence, changes related to certain ion-channel-encoding gene variants that have been observed in experiments can be directly attributed to a change of one or more parameters of these models. The Hay and Almog neuron models are based on electrophysiological recordings and cell stainings from rat neocortical slices, while the Kharche and Severi models are based on mouse and rabbit data, respectively. For details on the models, see Supplementary information.

Both L5PC models are simulated using NEURON software and Python interface using adaptive time-step integration. The 0-dimensional (point-cell) and 1-dimensional (chain of cells) SANC models are simulated using MATLAB, and the numerical integration is carried out using the variable time-step, stiff differential equation solver ode15s (0-dimensional) or the finite difference method with 0.01 ms time step (1-dimensional). For the 2-dimensional problem, we use the monodomain model^24^, which is simulated using the finite element method solver FEniCS^25^. For details on the spatial distribution of parameters in the 2D simulations, see ref.^26^. Scripts for running L5PC and 0D SANC simulations are publicly available (https://senselab.med.yale.edu/ModelDB/showModel.cshtml?model=187615).

### Genes included in the study

We chose the set of SCZ-associated genes as follows: We used the SNP-wise *p*-value data of ref. 2, and for each gene of interest, determined the minimum *p*-value among those SNPs that were located in the considered gene. We performed this operation for all genes encoding either subunits of voltage-gated Ca^2+^, K^+^, or Na^+^ channels, subunits of an SK, leak, or hyperpolarization-activated cyclic nucleotide-gated (HCN) channel, or Ca^2+^-transporting ATPases. Several of these genes were found to contain SNPs bearing a high risk of SCZ (*p*-value smaller than 3 × 10^*−*8^ in the data of ref. 2, namely, *CACNA1C*, *CACNB2*, *CACNA1I*, *ATP2A2*, and *HCN1*. Using a more relaxed threshold (*p*-value smaller than 3 × 10^*−*5^) extended this set by the genes *CACNA1D*, *SCN1A*, *SCN9A*, *KCNN3*, *KCNS3*, *KCNB1*, *KCNMA1*, and *ATP2B2*. This selection was identical to that in our previous work^16^. In this work, however, we concentrate on the genes that are likely to play a role in both L5PCs and SANCs: these are *CACNA1C*, *CACNB2*, *CACNA1I*, *ATP2A2*, *HCN1*, *CACNA1D*, and *SCN1A*.

It should be noted that we used the SNPs reported in ref. 2 only to name the above SCZ-related genes, and due to lack of data on their electrophysiological effects, we could not include the actual SCZ-related SNPs. In fact, only 14 of 527 SNPs that had a *p*-value smaller than 3 × 10^*−*5^ were highlighted in online databases (PubMed or SNPedia), and none of these 14 SNPs have yet been studied functionally, either in native or in heterologous cells. Therefore, we searched in PubMed for functional genomic studies reporting the effects of *any* genetic variant of the above genes, as described below. We only included studies that reported electrophysiological or intracellular Ca^2+^ imaging data, but we accepted studies performed using all tissue types. Nevertheless, most of the included studies were carried out in embryonic kidney cells.

### Gene variants and their downscaled versions

Table 1 lists all studies^27–56^ that we found where the effects of a variant were measured in a way that could be directly implemented as a parameter change in our models. A more detailed version of this table is given in Supplementary information, Supplementary Table S1. As SCZ is a polygenic disorder, it has been proposed that the disorder will not be induced by any of the SCZ-related SNPs alone, but only when sufficiently many of them are represented. Furthermore, as most of these SNPs are common variants^57^, it is likely that none of them alone can cause radical cardiac dysfunction—however, their combination could underlie the risk of heart disease that has been observed in SCZ patients. This paradigm was used in this study in a similar fashion as in ref. 16. If the variants described in Supplementary Table S1 altered the neural response or cardiac pacemaking too dramatically (see conditions A1–A5 and B1–B2 in Supplementary information), the changes in the model parameters were brought closer to zero (all in proportion) until a point *c* ∈[0, 1] where one or more of the scaling conditions were first violated. This downscaling was performed so that those parameters that may receive both negative and positive values were scaled linearly (∆ → *c*∆, where ∆ denotes the original increment to the underlying parameter as obtained from the literature data) while the parameters that receive only positive values were scaled on the logarithmic scale (Γ → Γ^*c*^, where Γ denotes the original factor of the underlying model parameter). In other words, the differences in offset and reverse potentials (*V*_offm_, *V*_offh_) between control and variant neuron were expressed as an additive term ( ± *x* mV), and this term *x* was multiplied by a parameter *c* in the downscaling procedure. By contrast, the differences in all the other model parameters (*V*_slo_, *τ*, *P*_up_, *γ*) between control and variant neuron were expressed as a multiplication (× *x*), where the downscaling caused this factor *x* to be exponentiated by the same parameter *c*.

**Table 1:** Table of the genetic variants used in this study

Gene	Refs.	Type of variant	Cell type
*CACNA1C*	^27^	L429T, L434T, S435T, S435A, S435P	TSA201
*CACNA1C*	^27^	L779T, I781T, I781P	TSA201
*CACNA1C*	^28^	G432X, A780X, G1193X, A1503X	TSA201
*CACNA1C*	^29^	I781X, C769P, G770P, N771P, I773P, F778P, L779P, A780P, A782P, V783P	TSA201
*CACNA1C*	^30^	I781T, N785A, N785G, N785L	TSA201
*CACNA1C*	^31^	Splice variants a1C77-A, -B, -C, and -D	TSA201
*CACNA1D*	^32,33^	Splice variant 42A	TSA201/HEK293
*CACNA1D*	^32,33^	Splice variant 43S	TSA201/HEK293
*CACNA1D*	^34,35^	Homozygous knockout	AV node cells / chromaffin cells
*CACNA1D*	^36^	A749G	TSA201
*CACNA1D*	^37^	V259D, I750M, P1336R	TSA201
*CACNA1D*	^38^	rCav1.3scg variant and related mutants	TSA201
*CACNB2*	^39^	T11I	TSA201
*CACNB2*	^40^	A1B2 vs A1 alone	HEK293
*CACNB2*	^41^	Splice variants N1, N3, N4, N5	HEK293
*CACNB2*	^42^	D601E	TSA201
*CACNA1I*	^43^	Alternative splicing of exons 9 and 33	HEK293
*CACNA1I*	^44^	Truncated cDNAs L4, L6, and L9	HEK293
*ATP2A2*	^45,46^	Heterozygous null mutation	Myocytes, embryonic stem cells
*ATP2A2*	^47^	Dairier’s disease related mutants	HEK293
*ATP2A2*	^48^	Dairier’s disease related mutants	HEK293
*SCN1A*	^49^	Q1489K	Cultured neocortical cell
*SCN1A*	^50^	L1649Q	TSA201
*SCN1A*	^51^	R859H	TSA201
*SCN1A*	^51^	R865G	TSA201
*SCN1A*	^52^	T1174S	TSA201
*SCN1A*	^53^	M145T	TSA201
*HCN1*	^54^	D135W, D135H, D135N	HEK293
*HCN1*	^55^	E229A, K230A, G231A, M232A, D233A, S234A, E235G, V236A, Y237A, EVY235-237DDD	Oocytes
*HCN1*	^56^	WAG-HCN1	Oocytes

For more details, see Supplementary Table S1 and Supplementary Table S2

The scaling conditions were designed such that the variant L5PCs and SANCs retain their baseline firing (L5PC) and pacemaking (SANC) behavior: Conditions A1–A3 require that the variant L5PCs respond with the same numbers of spikes to certain stimuli as the control L5PCs, while conditions A4 and B1 require that the firing (L5PC) or pacemaking (SANC) frequency is not radically changed, and conditions A5 and B2 make sure that the shape of the action potentials is not too different from that of the control cell.

The downscaling was done separately for each applied model; see Supplementary Table S2 for the scaling parameters *c* obtained for each variant and each model. Supplementary Fig. S1 illustrates the distribution of the variant effects of a single gene, *SCN1A*, on activation and inactivation voltage-dependency parameters in the Hay model, and shows that the variants span a large array of possible alterations of ion-channel dynamics. This is true also for the variants of other ion-channel-encoding genes (data not shown).

In the following, we present simulation data from the cells implemented with variants of different magnitude and direction, parametrized by variable *ϵ*. This parametrization is done so that the final effect sizes of the variants are the values of Supplementary Table S2 multiplied or exponentiated by *ϵc* (see Supplementary information). Variants with $\epsilon {\mathbf{ = }}\frac{1}{2}$ and $\epsilon {\mathbf{ = }}\frac{1}{4}$ mean that the variant effects on model parameters are half or quarter, respectively, of those of the threshold variants—these variants are, therefore, confirmed to obey the above-mentioned scaling conditions. In addition, we consider the variants $\epsilon {\mathbf{ = }} - \frac{1}{2}$ and $\epsilon {\mathbf{ = }} - \frac{1}{4}$, which represent parameter changes that are opposite to those of $\epsilon {\mathbf{ = }}\frac{1}{2}$ and $\epsilon {\mathbf{ = }}\frac{1}{4}$ variants.

## Results

### Pleiotropic effects of Na^+^ and non-selective ion channel gene variants are analogous

**Fig. 1:**
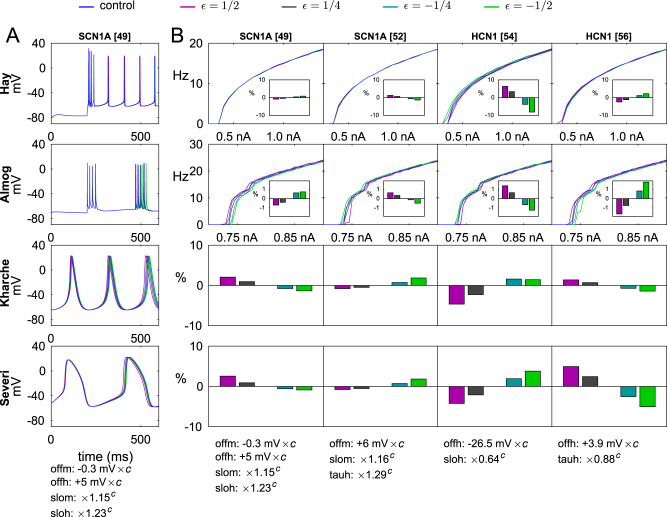
Effects of Na^+^ and HCN channel gene variants on L5PC and SANC excitability are qualitatively similar **a** The membrane-potential time courses of the modeled cells in control cells and Na^+^ channel variant cells. The L5PCs are stimulated with a somatic DC of amplitude 1.0 nA (Hay model, top panel) or 0.8 nA (Almog model, second from top) to induce stable spiking, while the SANCs rhythmically fire cardiac action potentials without any external stimulus (Kharche model third from top, Severi model on the bottom panel). Different colors represent different scalings of the same variant, parametrized by variable $\epsilon$. Variants with $\epsilon {\mathbf{ = }}\frac{1}{2}$ and $\epsilon {\mathbf{ = }}\frac{1}{4}$ mean that the variant effects on model parameters are half or quarter, respectively, of those of the threshold variants (see threshold parameters *c* in Supplementary Table S2)—these variants are, therefore, confirmed to obey the above-mentioned scaling conditions. In addition, we consider the variants $\epsilon {\mathbf{ = }} - \frac{1}{2}$ and $\epsilon {\mathbf{ = }} - \frac{1}{4}$, which represent parameter changes that are opposite to those of $\epsilon {\mathbf{ = }}\frac{1}{2}$ and $\epsilon {\mathbf{ = }}\frac{1}{4}$ variants. Blue: control, magenta:$\epsilon {\mathbf{ = }}\frac{1}{2}$, gray: $\epsilon {\mathbf{ = }}\frac{1}{4}$, cyan: $\epsilon {\mathbf{ = }} - \frac{1}{4}$, green: $\epsilon {\mathbf{ = }} - \frac{1}{2}$. **b** The f–I curves and pacemaking rhythms of the modeled cells for different Na^+^ channel and HCN channel variants. The L5PCs are stimulated with a somatic DC of amplitude ranging from 0.25 to 1.4 nA (Hay model) or 0.7 to 0.9 nA (Almog model), and the firing frequency is plotted against the stimulus amplitude. The insets show the change in threshold current at rest in relation to that of the control neuron (0.350 nA in the Hay model, 0.418 nA in the Almog model: note that the threshold for inducing single spikes at rest is lower than the threshold for inducing continuous spiking). For SANCs, the relative difference from control cell pacemaking rhythm (4.76 Hz in the Kharche model, 2.90 Hz in the Severi model) are shown. Apart for the varied effects of Na^+^ channel variants on L5PC f–I curves, it can observed that those variants that increase the L5PC firing frequency also mostly increase the pacemaking rhythm in SANCs, and vice versa

To characterize the joint implications of SCZ-related genes on neuron excitability and cardiac pacemaking, we started by analyzing the effects of the downscaled versions of genetic variants of Na^+^ and hyperpolarization-activated cyclic nucleotide-gated (HCN) channel-encoding genes, namely, *SCN1A* and *HCN1*. Fig. 1a shows the time course of the membrane potential for several versions of a *SCN1A* variant, predicted by both L5PC models (when a somatic DC stimulus is applied) and both SANC models (at steady pacemaking). Moreover, Fig. 1b shows the f–I curves, where the firing frequency is plotted against the amplitude of the injected current, and threshold currents for inducing a spike in an L5PC for several *SCN1A* and *HCN1* variants, and the changes these variants cause to the pacemaking frequencies in SANCs.

The results of Fig. 1 show that the changes in Na^+^ or HCN channels mostly caused similar effects in L5PCs and SANCs in terms of excitability. If the variant effect was excitatory (lower threshold of firing or steeper slope of f–I curve, i.e., higher gain) in L5PCs, usually the effect in SANCs was excitatory as well (faster pacemaking). We confirmed this observation by changing only one model parameter at a time (see Supplementary Fig. S2). However, the effects of Na^+^ channel variants on steady-state firing of the Hay-model neuron were usually small and often opposite to the corresponding effects in the Almog-model neuron. Exceptions to the above-mentioned trend arise also in HCN channel variants that affect both the threshold and slope of activation, leading to diverse effects in the two L5PC models (see Supplementary information).

### Pleiotropic effects of variants of Ca^2+^ channel or transporter-encoding genes are non-analogous

**Fig. 2:**
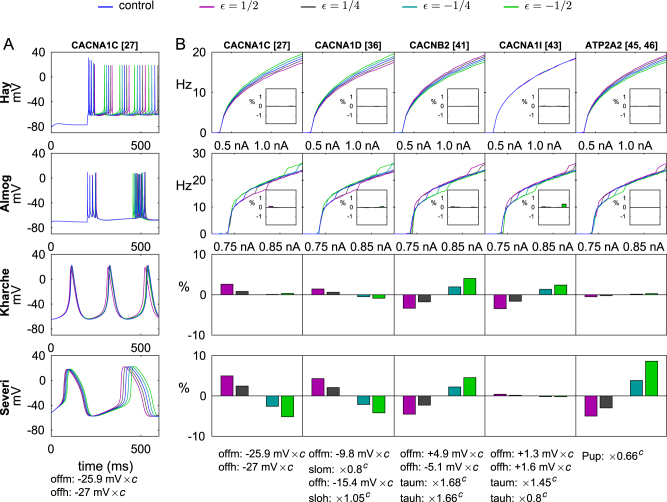
Ca^2+^ channel gene variants typically have opposite effects on L5PC firing and SANC pacemaking **a** The membrane potential time courses in control cells and cells implemented with a *CACNA1C* variant (the first variant in Supplementary Table S2), see Fig. 1a. **b** The f–I curves and pacemaking rhythms of the modeled cells implemented with a Ca^2+^ channel or transporter variant, see Fig. 1b. Apart from the *CACNA1I* variants that show mixed effects, it can be seen that those variants that increase the pacemaking rhythm in SANCs, decrease the L5PC firing frequency, and vice versa. Variant effects on threshold currents in L5PCs are generally small

**Fig. 3:**
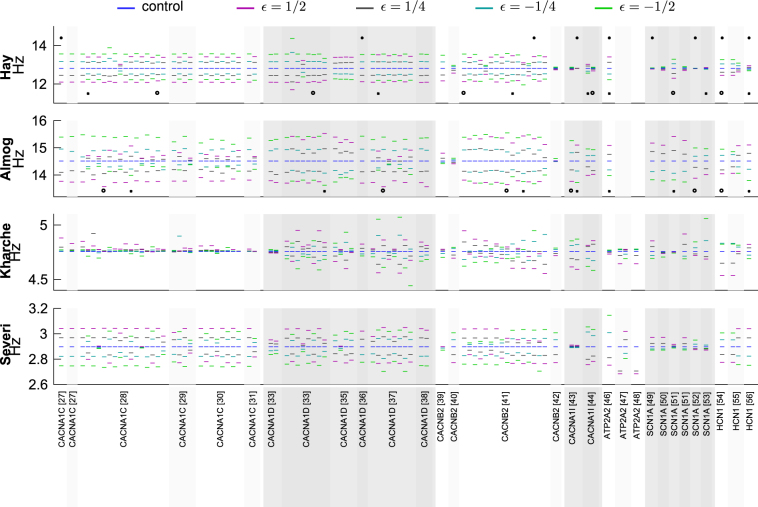
Overview of variant effects on L5PC firing and SANC pacemaking The average firing frequencies and pacemaking rhythms are shown for all variants of Supplementary Table S2. Top panel: Firing rates in the Hay model, averaged over stimulus amplitudes 0.35–1.4 nA. Second panel: Firing rates in the Almog model, averaged over stimulus amplitudes 0.7–0.9 nA. Third panel: Pacemaking rates in the Kharche model. Bottom panel: Pacemaking rates in the Severi model. See Figs. 1 and 2 for more detailed data. Variants shown in Figs. 1 and 2 are highlighted with asterisks, and variants used in combinations of Supplementary Fig. S10 are highlighted with crosses ('* × *') and circles (' ∘ ')

Next, we analyzed the implications of the genes that encode subunits of voltage-gated Ca^2+^ channels (*CACNA1C*, *CACNA1D*, *CACNB2*, *CACNA1I*) or Ca^2+^ transporters (*ATP2A2*). In Fig. 2, a representative set of variants of these genes is picked, and their effects on L5PC and SANC behavior is illustrated. In a similar fashion as in Fig. 1, Fig. 2a shows the time course of the membrane potential for different versions of one variant, and Fig. 2b shows the f–I curves and pacemaking frequencies for several variants. A general trend is that variants that increase the pacemaking rhythm in SANCs decrease the firing frequency in L5PCs, and vice versa. This is supported by Fig. 3 showing the mean (averaged over the stimulus amplitudes of Fig. 1) firing frequencies of all implemented variants for the L5PC models and the corresponding pacemaking frequencies for the SANC models, and by Supplementary Figs. S3 and S4 showing results from single-parameter variants. The average firing rates and pacemaking rates of Ca^2+^ channel and transporter variants were anticorrelated with a correlation coefficient −0.53 to −0.79, while the corresponding correlation coefficients for Na^+^ and HCN channel variants were 0.47–0.78 (see Table 2). Exceptions to this trend are discussed in Supplementary information, and the reasons for them are illustrated in Supplementary Figs. S5, S6, S7, and S8, where the time courses of different current species are plotted for control and variant neurons.

**Table 2:** Firing frequencies of L5PCs and pacemaking frequencies of SANCs are correlated for Na^+^ channel and *HCN1* variants, but anticorrelated for Ca^2+^ channel and Ca^2+^ transporter variants

(A) Na^+^ and HCN channel variants		
	Hay	Almog
Kharche	0.4965	0.7477
Severi	0.5031	0.7818

(B) Ca^2+^ channel and transporter variants		
	Hay	Almog
Kharche	−0.5168	−0.5971
Severi	−0.7700	−0.7905

(C) All variants				
	Hay	Almog	Kharche	Severi
Hay	1	0.6896	−0.4364	−0.7223
Almog	0.6896	1	−0.4233	−0.6501
Kharche	−0.4364	−0.4233	1	0.6645
Severi	−0.7223	−0.6501	0.6645	1

The table shows the correlation coefficients between the firing or pacemaking frequencies of $\epsilon {\mathbf{ = }} - \frac{1}{4}$ variants (see Fig. 3), as predicted by the different models. **A**: Only data from *SCN1A* and *HCN1* variants included. **B**: Only data from *CACNA1C*, *CACNA1D*, *CACNB2*, *CACNA1I* and *ATP2A2* variants included. **C**: All variants included. The variants that were not applicable for L5PC models (see the Supplementary Table S2 entries corresponding to studies^47,48^) were omitted. The relatively strong anticorrelations between L5PC and SANC model data in (C) reflect the fact that majority (80) of the variants (94 in total) operated on Ca^2+^ channels and transporters

The difference in variant effects between L5PC and SANC models is caused by differences in downstream effects of the Ca^2+^ currents. All four models describe certain aspects of how the intracellular [Ca^2+^], increased by the current influx through the Ca^2+^ channels, affects the function of other transmembrane ion channels or exchangers. In the L5PC models, increased intracellular [Ca^2+^] activates the Ca^2+^-dependent K^+^ channels, i.e., the SK channels (and BK channels in the Almog model) that are hyperpolarizing. These channels are traditionally absent from the SANC models, and while recent evidence suggests they may contribute to sinus-node electrophysiology^58,59^, a well-recognized characteristic of SANC function is that enhanced Ca^2+^ cycling is an important contributor to increased pacemaking frequency both *ex vivo* and *in vivo* ^60^.

### Changes in SANC excitability affect signal propagation

**Fig. 4:**
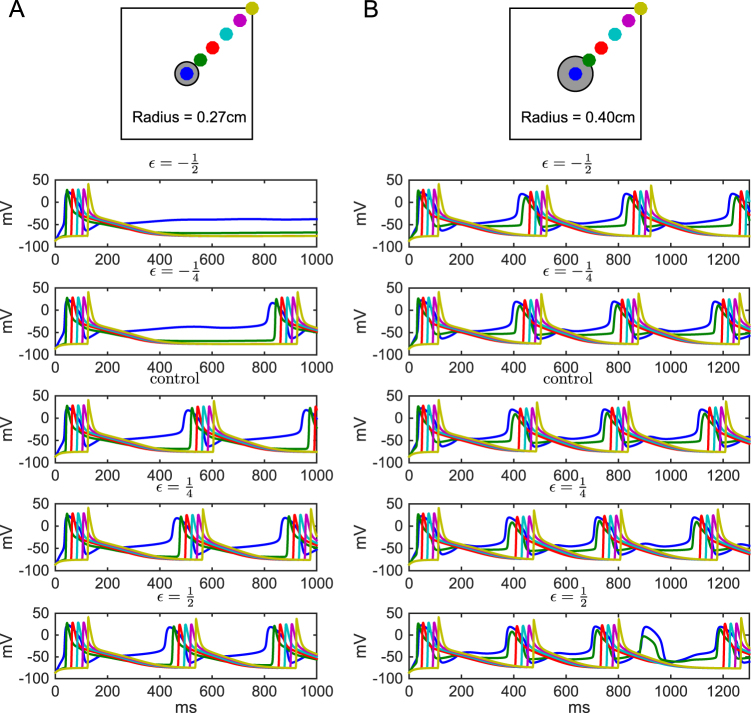
Signal propagation from SAN to surrounding atria is altered in a Ca^2+^ channel variant A tissue of size 3 × 3 cm was modeled using a diffusion constant of 1.2 cm^2^/s in the direction of the monitored fiber and 0.25 cm^2^/s in the direction orthogonal to this. A spatial resolution of 151 × 151 nodes (0.02 cm) and temporal resolution of ∆*t* = 0.125 ms was used. The panels show the membrane potential as a function of time at six different locations of the modeled tissue for different scalings of a *CACNA1C* variant (the first variant of Supplementary Table S2). The center (blue) expresses membrane potential dynamics similar to that seen in single-cell experiments (Figs. 1 and 2), while near the borders (magenta and yellow), the action potentials are sharper and more alike to those presented in ref.^62^. The left panels (A) show the results in a model tissue with a smaller (radius 0.27 cm) SAN, and the right panels (B) show the results using a larger (radius 0.40 cm) SAN. In the variants with smallest effect ($\epsilon {\mathbf{ = }} - \frac{1}{4}$ and $\epsilon {\mathbf{ = }}\frac{1}{4}$), only the pacemaking frequency is altered, but in other variants, more radical phenotypes are observed. The $\epsilon {\mathbf{ = }} - \frac{1}{2}$ variant ceases pacemaking in the tissue with smaller SAN, and in the simulations with the larger SAN, all action potentials initiated by the $\epsilon {\mathbf{ = }}\frac{1}{2}$ variant SANCs are not successfully transmitted to the atrial tissue

We implemented a simple 1-dimensional model of interconnected SANCs to analyze the effects of the variants on signal conduction. The SANCs were identical, and were connected to each other with a diffusion constant of 6  × 10^4^ µm^2^/ms (as in ref. 61). In this 1-dimensional model, the SANC components were first voltage-clamped to a hyperpolarized membrane potential (−64 mV), and then a fraction of them was clamped to a depolarized membrane potential ( + 23 mV), and the conduction of this pulse of activation across the SANCs could be observed. Supplementary Fig. S9 shows that subtle variants of Ca^2+^ channel genes could slightly affect the signal conduction velocity along the sinoatrial node (SAN), and an overview of results for all variants is shown in Supplementary Fig. S11. This propagation is altogether rapid, and, therefore, unlikely to meaningfully alter the speed or sequence of cardiac activation. However, these subtle changes may have larger effects in multiple dimensions and particularly in determining the ability of the SAN to provide sufficient current to activate the surrounding tissue. To test this hypothesis, we applied our variants in a 2-dimensional model including SAN and surrounding atrial tissue. In this experiment, we modeled the SAN tissue using the Severi model and the atrial tissue using the model of ref. 62. We implemented the *CACNA1C* variant^27^ (the first entry of Supplementary Table S2) in the SAN tissue using the scaling threshold parameter *c* = 0.102. Figure 4 shows the initiation and propagation of cardiac action potentials in this composite 2-dimensional model tissue both for control and variant cases. An interesting finding is shown in Fig. 4b, where the $\epsilon {\mathbf{ = }}\frac{1}{2}$ variant caused failure of a premature SAN beat to propagate into the atrial tissue when a large SAN (radius *r* = 0.4 cm) was used. This in turn resulted in an increased beat-to-beat interval, which if frequent enough, would manifest as increased R-R variability and could be clinically identified as an SAN dysfunction.

Figure 4a shows that when a small SAN (radius *r* = 0.27 cm) was combined with the $\epsilon {\mathbf{ = }} - \frac{1}{2}$ variant, spontaneous SAN activity was silenced. In this case the electrotonic load of the surrounding atrial tissue prevents the current generated by the SANCs from being sufficient to generate a propagating depolarization wave, and thus pacemaking ceases. Such a sharp loss of sinus excitation would be associated with severe (class 1) SAN dysfunction. In an intermediate-sized SAN (radius *r* = 0.34 cm), by contrast, all variants show a stable pacemaking (data not shown).

## Discussion

In this work, using computational modeling we showed how subtle genetic variants of SCZ-associated genes can cause comorbid effects in neuronal and cardiac function. We used models of L5PCs and SANCs due to the biological significance of these cells (in cortical information processing and heart beat initiation, respectively), the high level of biophysical detail with which they are described, and the similarities in expression of the genes studied here. We showed that small changes in the parameters governing the voltage-dependence and time constants of activation and inactivation of different ion channels caused observable effects in both L5PC and SANC function (Figs. 1–3). In the case of Ca^2+^ channel gene variants, these changes typically had opposite effects on cell excitability in L5PCs compared to SANCs (higher L5PC firing frequency ↔ lower SANC pacemaking frequency, see Fig. 2), while in the case of Na^+^ or HCN channel variants, the effects were mostly similar (higher L5PC firing ↔ frequency higher SANC pacemaking frequency, see Fig. 1). Our framework is well suited to studying polygenic effects, which is especially important in SCZ: we showed that combinations of subtle variants of different genes can have a large effect on cell excitability (Supplementary Fig. S10). These results are, to our knowledge, the first findings from a polygenic analysis of the pleiotropic effects of genetic variants on neural and cardiac functions.

Our result showing that the gain-of-function Ca^2+^ channel variants (i.e., variants that increase Ca^2+^ currents) increase the excitability of SANCs but decrease the excitability of L5PCs, is in line with previous studies. For SANCs, early indirect experimental evidence (e.g., in ref. 63) indicated that larger Ca^2+^ currents (mediated by an increase in extracellular [Ca^2+^]) caused faster SAN pacemaking. More recent studies showed that a variety of manipulations that increase whole-cell Ca^2+^ load also increase SANC pacing frequency, and that their common mechanistic link is spontaneous sarco-endoplasmic reticulum Ca^2+^ release, which accelerates early SANC depolarization due to Na^+^-Ca^2+^ exchange^60^. The source of SANC pacing is, however, still under debate^64^. The contexts and molecular players determining the importance of these intracellular Ca^2+^ fluxes remain hotly debated, but there is little doubt that gain-of-function effects in SANC Ca^2+^ channels results in more rapid beating due either to direct depolarization or secondarily to Ca^2+^ release from the sarco-endoplasmic reticulum.

For L5PCs, there are numerous experimental studies analyzing the medium-duration after-hyperpolarization (mAHP) current, which is mediated by SK channels and is strong in L5PCs^65^, but its Ca^2+^ channel-dependent inhibitory effect on neuron firing has rarely been compared with the direct excitatory effect of the Ca^2+^ channels. In ref. 66, the effect of blockade of L-type Ca^2+^ channels on EPSP amplitude was non-significant (albeit slightly weakening the excitability) compared to control. By contrast, computational studies of L5PCs repeatedly predicted that a decrease in current through Ca^2+^ channels increase the L5PC excitability (due to the consequent decrease in Ca^2+^-dependent K^+^ current), and vice versa. In addition to the present work, this was concluded in our earlier work^16^ and in an independent study employing a network of L5PCs^67^, where the blockade of voltage-gated Ca^2+^ channels (especially of those located at the soma) increased the network excitability. Confirming and extending these results may require a spatially detailed neuron model and an extended description of Ca^2+^ dynamics (cf.^65,68^).

Apart from L5PCs, the SK-Ca^2+^ current coupling that reverses the output gain of the Ca^2+^ currents has been experimentally observed in other types of neurons^69^. These data could be used to validate the results obtained from our method when applied to other cell types. L5PCs are of particular interest among the types of neuron in the brain due to their role as an integrator of sensory feed-forward and cortical feed-back information^17^. However, future work should address these research questions in other types of neurons as well, as made possible by the increasing availability of biophysical neuron models^70^, and models of other pacemaker cells and myocytes in the heart.

The differences in the levels of biophysical detail between the applied models prevent a consistent use of some of our variants. Ca^2+^ dynamics were described in more detail in the SANC models than in the L5PC models, which reflects the known importance of Ca^2+^ cycling in SANC function and allowed for more comprehensive analysis of SERCA (encoded by *ATP2A2*) variants and their effects on pacemaking. None of the models, however, takes into account nanoscale Ca^2+^ release events, which may crucially affect the cell electrophysiology^71^. The analyses of genetic effects of *ATP2A2* were restricted in L5PC models to one variant^45,46^, whose functional effects had previously been measured both in terms of SERCA uptake and cytosolic Ca^2+^ transients. Of these quantities, the effect on SERCA uptake could be directly applied to SANC models, while the effect on cytosolic Ca^2+^ transients could be applied to L5PC model parameters. Note, however, that the latter effect might be highly dependent on the cell type—the experiments of^46^ were carried out in myocytes. There is a trend toward the development of increasingly detailed biophysical neuron models and hence a L5PC model incorporating the functions of the endoplasmic reticulum (ER), SERCA pump, and relevant Ca^2+^ signaling molecules could be expected in the near future^72^. Using such models would mitigate the above-mentioned limitation in our approach.

The *I*_*f*_ current has notably different characteristics in the two SANC models. In the Kharche model, the description of the *I*_*f*_ current was formulated for *HCN4*-based currents, which have a very negative half-activation potential (−106.8 mV in the Kharche model) and a less steep slope (16.3 mV). By contrast, in the Severi model the *I*_*f*_ current had a much higher half-activation potential (−52.5 mV) and a steeper slope (9.0 mV). The choices concerning the *I*_*f*_ current in the Severi model are based on experiments made on rabbit SANCs where expression of both *HCN1* and *HCN4* were found^21,73,74^, while the Kharche model refers to experiments made on mouse SANCs where expression of only *HCN4* was observed^20,75^. However, other studies found *HCN1* expression also in mouse SANCs^76,77^—*HCN1* is also expressed in human SANCs^78^. Thus, the contribution of *HCN1* variants to SANC electrophysiology can be expected in both mice and humans, the latter contribution being an important assumption underlying our study.

Despite the differences in the voltage-dependencies of the *I*_*f*_ current inactivation, both SANC models agree on the fact that the current is most of the time depolarizing (reversal potentials were approximately −24 mV and −4 mV in the Kharche and Severi models, respectively). Moreover, the (depolarizing) amplitude of the *I*_*f*_ current is very similar in the two models (0.006 nA in Kharche model and 0.0067 nA in the Severi model, data not shown). Accordingly, the effects of the HCN channel variants on the SANC pacemaking were qualitatively similar in the two models, as shown in Figs. 1 and 3, and Supplementary Fig. S2 (an exception is the second-to-last variant of Fig. 3, whose effects on L5PC models were non-analogous as well, as discussed above).

Our framework is based on downscaling the electrophysiological effects of experimentally studied genetic variants, and as a theoretical approach, it has its limitations. It is not known whether the SCZ-associated SNPs in voltage-dependent ion channel-encoding genes have a measurable effect on the voltage-dependence and kinetics of the underlying channel or not. Furthermore, the magnitudes of the variant effects depend on the conditions used for downscaling the variants, and due to the non-linearity of the neuron models, these conditions may have to be adjusted for each operated model separately to ensure that a single (downscaled) variant does not totally change any fundamental aspect of the cell functionality. Nevertheless, the downscaling framework shows promise as a tool for studying interactions of modest genetic effects, and it can be extended to new cell types and classes of genes. Another limitation of our study stems from the biophysical details that are missing from our models. Like the vast majority of the neuron and cardiac cell models of today, our models do not allow examining the effects of certain biological phenomena controlling the function of ion channels, such as phosphorylation, spatial affinity, or heterogeneous subunit compositions. Extending the models to capture some of these biophysical details would be useful especially in the study of SCZ, as the risk of SCZ has been associated with alterations in not only genes controlling intrinsic electrogenesis (voltage-gated ion channels) and neurotransmission (synaptic ion channels), but also those controlling the calcium signaling machinery affecting the two through protein phosphorylation^79^. Meanwhile, novel tools in neuroinformatics, such as the automated large-scale classification of ion channel models as presented in ref. 80, could help in comparing the modeled effects of genetic variants between different neuron models and making predictions of the cellular functions in modified baseline conditions such as altered temperatures.

Our results shed light on the correlations between neuronal and cardiac phenotypes in SCZ patients. Due to the complexity of clinical manifestations of SCZ, the neural underpinnings of the symptoms are not well understood, but there is a generic hypothesis of SCZ being a disorder of cortical excitability^81,82^. To this end, altered L5PC excitability has been proposed as a contributor to the observed SCZ symptoms and phenotypes, such as hallucinations^16,17^. Altered synaptic function has also been associated to SCZ (suggested both by GWASs and imaging studies), but is out of the scope of the present study. The L5PC functions studied here were restricted to responses to somatic DC—for a more detailed analysis of the effects of the variants on integration of synaptic inputs, we refer to our earlier work^16^. In this work, we showed that the same subtle genetic variants that altered the L5PC excitability, also altered SANC pacemaking frequency and rate of propagation of the cardiac action potential. While these deviations are specific to pacemaker tissues in the heart, similar pleiotropic effects occurring in the ventricular myocardium could prolong the action potential or increase dispersion of repolarization, and thus be associated with the prolonged QT interval observed in drug-free SCZ patients^83^. An interesting observation is that the variants may affect the probability of successful signal propagation from the SAN to the surrounding atrial tissue, as shown in Fig. 4b. In these simulations, the variant effects were only implemented in the sinoatrial tissue—were they present also in the atrial tissue, the observed effects on signal propagation could be larger. When this propagation failure is complete, the condition is termed third degree sinoatrial block. More subtle effects include a sporadic block that simply alters P-P variability (and therefore R-R variability) similar to that shown in Fig. 4b. By inducing irregular long pauses in ventricular activation, this type of behavior may contribute to the emergence of ventricular tachycardias, particularly the torsade de pointes that accompanies both acquired and congenital long QT syndrome^84,85^. This form of polymorphic ventricular tachycardia is also associated with the drug-induced long QT syndrome that is a major contraindication of many antipsychotic drugs^9^.

Our results provide interesting views on the dual effects of SNP-like effects on neuronal and cardiac excitability. While the rare variants of ion-channel-encoding genes often have a disabling or even life-threatening phenotypic effect, the effects of common variants may be subtle and highly specific to certain types of tissue or cell type. The work at hand illustrates the polygenic effects of SCZ-associated genes by borrowing the functional genomic data from gene variants that implicate other, typically larger, phenotypic consequences, and studying the cellular functions under downscaled variant effects. These results provide an important viewpoint on the polygenic alterations of neuronal and cardiac excitability, but eventually, the electrophysiological consequences of the common, SCZ-associated SNPs should be assessed as well. Novel automated cell-patching methods^86,87^ could help in this vast task.

To conclude, the current findings support the use of a polygenic mathematical modeling approach to understand more of the pathobiology related to the GWAS-revealed SCZ-associated loci. Our results suggest overlapping but non-identical mechanisms through which subtle SNP-like variants of ion-channel and calcium-transporter-encoding genes modulate the intrinsic excitability of neurons and heart cells. This may explain some of the comorbidity between cardiac disease and SCZ, and could facilitate development of antipsychotic drugs with fewer cardiac side-effects.

## Electronic supplementary material

Supplementary material
