# Supplementary material for: Pleiotropic effects of schizophrenia-associated genetic variants in neuron firing and cardiac pacemaking revealed by computational modeling
Source: Transl Psychiatry. 2017 Nov 17;7:5. doi: 10.1038/s41398-017-0007-4 (PMC5802468; doi:10.1038/s41398-017-0007-4)
Supplement: Supplementary file 1 — Supplementary material [file 41398_2017_7_MOESM1_ESM.pdf]

# Pleiotropic effects of schizophrenia-associated genetic variants in neuron firing and cardiac pacemaking revealed by computational modeling. Supplementary information

## Methods

### The L5PC models and underlying ion channels

Both neuron models that we employ, the "Hay model" [S1] and the "Almog model" [S2], are multicompartmental Hodgkin-Huxley type of models with reconstructed layer V thick-tufted pyramidal neuron morphologies. The Hay model includes the following ionic currents: Fast inactivating $Na^+$ current ($I_{Nat}$), persistent $Na^+$ current ($I_{Nap}$), non-specific cation current ($I_h$), muscarinic $K^+$ current ($I_m$), slow inactivating $K^+$ current ($I_{Kp}$), fast inactivating $K^+$ current ($I_{Kt}$), fast non-inactivating $K^+$ current ($I_{Kv3.1}$), high-voltage-activated $Ca^{2+}$ current ($I_{CaHVA}$), low-voltage-activated $Ca^{2+}$ current ($I_{CaLVA}$), small-conductance $Ca^{2+}$-activated $K^+$ current ($I_{SK}$), and the passive leak current ($I_{leak}$). The Almog model includes a slightly different set of ionic currents: Fast inactivating $Na^+$ current ($I_{Nat}$), non-specific cation current ($I_h$), slow inactivating $K^+$ current ($I_{Kp}$), fast inactivating $K^+$ current ($I_{Kt}$), high-voltage-activated $Ca^{2+}$ current ($I_{CaHVA}$), medium-voltage-activated $Ca^{2+}$ current ($I_{CaMVA}$), small-conductance $Ca^{2+}$-activated $K^+$ current ($I_{SK}$), large-conductance voltage and $Ca^{2+}$-gated $K^+$ current ($I_{BK}$), and finally, the passive leak current ($I_{leak}$). The main difference between the two models, apart from the different set of modeled ion channels, is that in building the Almog model, the authors allowed the maximal ion-channel conductances to vary spatially, leading to a fit with optimal spatial distribution (usually piece-wise linear) along the dendrites [S2]. Consequently, in the Almog model a "hot zone" of $Ca^{2+}$ channels is missing, while in the Hay model it is assigned along the apical dendrite at a distance from 685 to 885 μm from the soma.

To date, there is no clear consensus on which particular ion channel subunits underlie each of these currents in L5PCs. mRNAs of ion channel-encoding genes *KCNA2*, *KCND2*, *KCND3*, *CACNA1A*, *CACNA1B*, *CACNA1C*, *CACNA1D*, *CACNA1E*, *CACNA1G*, *CACNA1H*, *CACNA1I*, *HCN1*, and *HCN2* were observed in L5PCs in a study of postnatal rat neocortices at different stages of development [S3]. Of these, *CACNA1A*, *CACNA1B*, *CACNA1C*, and *CACNA1D* are known to contribute to $I_{CaHVA}$, and *CACNA1G*, *CACNA1H*, and *CACNA1I* contribute to $I_{CaLVA}$ and possibly $I_{CaMVA}$[1], while the genes *KCNA2*, *KCND2*, and *KCND3* might contribute to the slow $I_{Kp}$ current. Expression of *KCNC1* was confirmed in certain subpopulations of L5PCs [S5], and expression of *KCNB1* and *KCNB2* has been observed in layer V or VI pyramidal neurons [S6]. The slow activation kinetics of channels composed of *KCNB1* and *KCNB2* subunits makes them likely contributors to the $I_{Kp}$ current, while *KCNC1*-based channels form the $I_{Kv3.1}$ current included in the Hay model. Expression of muscarinic potassium channel-encoding genes *KCNQ2* and *KCNQ3* was observed in L5PCs in [S7], and these genes are known to contribute to the $I_m$ current.

Expression of sodium channel subunit-encoding genes *SCN2A* and *SCN8A* was observed in pyramidal cells of all layers of human epileptic tissue [S8]. In another study, the genes *SCN1A*, *SCN2A*, *SCN3A*, and *SCN6A* were found to be expressed in L5PCs [S9], while expression of *SCN1A* was not observed in [S8]. Of the genes encoding sodium $\alpha$ subunits, *SCN1A*, *SCN2A* and *SCN3A*, alongside with *SCN9A*, are tetrodotoxin-sensitive [S10] and hence form both the transient ($I_{Nat}$) and persistent ($I_{Nap}$) $Na^+$ currents. Whether these genes contribute to $I_{Nat}$ or $I_{Nap}$ may depend on the modulatory subunits that they are associated with [S11]. Expression of *KCNN1* and *KCNN2* has been observed in L5PCs, while the expression of the third gene affecting the $I_{SK}$ current, *KCNN3*, is weak throughout the neocortex but more prominent in pyramidal neurons in CA1 [S12]. The $\alpha$ subunits of BK channels, encoded by *KCNMA1* and giving rise to the $I_{BK}$ current in the Almog model, were detected in L5PCs in [S13].

Most of the above genes code for the $\alpha$ subunit of the underlying ion channel. Many ion channels also incorporate modulatory subunits, the presence of which may change the kinetics or voltage-dependence of the ion channel. As an example, the $\beta 2$ subunit of $Ca^{2+}$ channels, encoded by *CACNB2*, associates with L-type $Ca^{2+}$ channels (where the $\alpha$ pore is encoded by *CACNA1S*, *CACNA1C*, *CACNA1D* or *CACNA1F*).

In addition to describing the dynamics of these ionic channels, the models also describe the dynamics of the intracellular $Ca^{2+}$ concentration, $[Ca^{2+}]_i$. According to the models, $[Ca^{2+}]_i$ is increased by the current flow through $Ca^{2+}$ channels, and is otherwise decreased towards a resting-state level of $[Ca^{2+}]_i$. This extrusion of $Ca^{2+}$ is contributed by many intracellular molecules, but two types of proteins possess a key role, namely, sarco/endoplasmic reticulum $Ca^{2+}$ ATPase (SERCA) and plasma membrane $Ca^{2+}$ ATPase (PMCA). The SERCA proteins pump intracellular $Ca^{2+}$ into sarcoplasmic or endoplasmic

[1] The molecular basis of LVA $Ca^{2+}$ currents in L5PCs is still disputed. Recent data show that T-type $Ca^{2+}$ currents are not present in the soma nor its neighbourhood [S4]. Therefore, the role of low or medium-voltage activated $Ca^{2+}$ channels was assigned to R-type channels ($\alpha$ pore encoded by *CACNA1E* gene) in the Almog model [S2]. In the Hay model, however, the LVA channel kinetics were based on measurements carried out on T-type $Ca^{2+}$ channels. In this work, we consider both the Almog-model $I_{CaMVA}$ current and the Hay-model $I_{CaLVA}$ current as a possible target of the T-type $Ca^{2+}$ channel gene *CACNA1I*.

reticulum, which later may release the excessive $Ca^{2+}$ to strengthen the $Ca^{2+}$ pulses entering through the voltage-gated $Ca^{2+}$ channels. SERCA pumps are encoded by genes ATP2A1, ATP2A2, and ATP2A3, of which ATP2A2 is widely expressed in the brain [S14]. PMCA proteins pump intracellular $Ca^{2+}$ into the extracellular medium, and are encoded by genes ATP2B1, ATP2B2, ATP2B3, and ATP2B4, all of which are widely expressed in the brain [S15, 16].

## The SANC models and underlying ion channels

We apply two SANC models, namely, the "Kharche model" [S17] and the "Severi model" [S18]. The Kharche model is based on experimental data collected from mouse SANCs, and includes the following transmembrane currents: $Na^+$ channel isoform Nav1.1 current ($I_{Na,1.1}$, $\alpha$ subunit encoded by *SCN1A*), $Na^+$ channel isoform Nav1.5 current ($I_{Na,1.5}$, $\alpha$ subunit encoded by *SCN5A*), L-type $Ca^{2+}$ channel isoform Cav1.2 current ($I_{CaL,1.2}$, $\alpha$ subunit encoded by *CACNA1C*), L-type $Ca^{2+}$ channel isoform Cav1.3 current ($I_{CaL,1.3}$, $\alpha$ subunit encoded by *CACNA1D*), T-type $Ca^{2+}$ current ($I_{CaT}$), rapid delayed rectifying $K^+$ current ($I_{Kr}$), slow delayed rectifying $K^+$ current ($I_{Ks}$), hyperpolarization-activated current ($I_f$), transient component of 4-AP-sensitive $K^+$ currents ($I_{to}$), sustained component of 4-AP-sensitive $K^+$ currents ($I_{sus}$), time-independent $K^+$ current ($I_{K1}$), sustained inward $Na^+$ current ($I_{st}$), $Na^+$ -$K^+$ pump current ($I_{NaK}$), $Na^+$ /$Ca^{2+}$ exchanger current ($I_{NaCa}$), background $Na^+$ current ($I_{b,Na}$), background $K^+$ current ($I_{b,K}$), and background $Ca^{2+}$ current ($I_{b,Ca}$).

The Severi model is based on data from rabbit SANCs, and includes the following transmembrane currents: $Na^+$ current ($I_{Na}$), L-type $Ca^{2+}$ current ($I_{CaL}$), T-type $Ca^{2+}$ current ($I_{CaT}$), rapid delayed rectifying $K^+$ current ($I_{Kr}$), slow delayed rectifying $K^+$ current ($I_{Ks}$), hyperpolarization-activated current ($I_f$), transient component of 4-AP-sensitive $K^+$ currents ($I_{to}$), acetylcholine-dependent $K^+$ current ($I_{K,Ach}$), $Na^+$ -$K^+$ pump current ($I_{NaK}$), and $Na^+$ /$Ca^{2+}$ exchanger current ($I_{NaCa}$).

The modeled $Na^+$ currents represent channels including one or both of the two major [S19] $Na^+$ channel $\alpha$ subunits in SANCs, Nav1.1 $\alpha$ (encoded by *SCN1A*, sensitive to tetrodotoxin) and Nav1.5 $\alpha$ (encoded by *SCN5A*, tetrodotoxin-resistant). The two modeled L-type $Ca^{2+}$ currents represent high-voltage-activated $Ca^{2+}$ channels that contain either a CaV1.2 $\alpha$ subunit (encoded by *CACNA1C*) or a CaV1.3 $\alpha$ subunit (encoded by *CACNA1D*). The T-type $Ca^{2+}$ current represents low-voltage-activated $Ca^{2+}$ channels, where the $\alpha$ subunit is encoded by *CACNA1G*, *CACNA1H* or *CACNA1I*. Of these, *CACNA1G* gene is much stronger expressed in SANCs than *CACNA1H* or *CACNA1I* [S20]. For the molecular basis of the rest of the currents, we refer to more detailed descriptions [S21].

In addition to the transmembrane currents, both models have a description of the intracellular $Na^+$ , $K^+$ , and $Ca^{2+}$ dynamics. The intracellular $Na^+$ and $K^+$ concentrations are described by single variables, but the intracellular $Ca^{2+}$ dynamics are considered in four separate subcellular compartments: network sarcoplasmic reticulum (NSR, part of sarcoplasmic reticulum, SR), junctional sarcoplasmic reticulum (JSR), sub-membrane space in the immediate vicinity to the membrane, and the rest of the intracellular medium. The $Ca^{2+}$ ions flow via the $I_{CaL}$ and $I_{CaT}$ currents from the extracellular medium to sub-membrane space, where the confined volume maintains a high local calcium concentration and thereby permits $Ca^{2+}$-dependent signalling between the surface membrane calcium channels and ryanodine receptors of the JSR. From this compartment, $Ca^{2+}$ is then extruded to the extracellular medium via electrogenic NaCa exchange current, $I_{NaCa}$. $Ca^{2+}$ ions also diffuse from the sub-membrane space to the bulk cytosol, from which they are resequestered into the network SR by the SERCA pump. From NSR, the $Ca^{2+}$ ions diffuse to JSR, which releases them to the sub-membrane space, closing the loop (see Figure 1 in [S17] and Figure 1 in [S18]).

## Functional genomics literature review

Many of the genes that are now confirmed to be linked to SCZ, have previously been shown to play a role in regulating the activation/inactivation kinetics of certain types of cellular transmembrane currents in animal or *in vitro* studies. Typically, these studies involved transfection of ion channel-encoding DNA into cells that normally do not express the considered ion channels, and documentation on how a variant DNA changed the electrophysiological properties of the cells compared to the cells transfected with control DNA. Moreover, many studies have demonstrated the effects of certain variants of a calcium signaling toolkit gene [S22] on the dynamics of the $Ca^{2+}$ concentration in the intracellular medium.

We searched through the literature on functional genomics for genes *CACNA1C*, *CACNA1D*, *CACNB2*, *CACNA1I*, *ATP2A2*, *SCN1A*, and *HCN1* to find data on how genetic variations change the ion channel behavior or intracellular $Ca^{2+}$ dynamics. Due to lack of data reported for a single animal and cell type, we included studies performed in various animal species and across different tissues. We concentrated on studies that fulfilled the following conditions:

- The study applied a genetic variant of one of the genes of interest.
- The properties of the cell expressing the gene variant were studied using electrophysiology or $Ca^{2+}$ imaging.
- The deviation between the variant cell property and the control cell property could be implemented in the applied L5PC and SANC models as a change of a model parameter values.
- The observed effect of the gene variant was not solely on the expression or ion channel density level.

The last condition, which ruled out studies where the effect was only shown on channel density, was set due to the multitude of pathways that may contribute to such an effect [S23]. By contrast, the effects on channel kinetics (activation and inactivation threshold potentials, sensitivity to membrane potential, and opening/closing time constants) were expected to be more straightforwardly dependent on the way these channels are genetically coded.

Table 1 shows all studies that were found to obey the above conditions. An expansion of this table is given in Table S1, added with details about the effects of each variant and the underlying experimental data.

Some of the studies cited in Table 1 considered several variant types, and in these cases, the range of possible effects on model parameters is considered (see Table S1). If the range spanned both increasing and decreasing effects, the analyses were carried out for both endpoints of the range, otherwise only the maximal deviation from the control value was considered. Also, in case one of the endpoints of the range was very close to the control value, namely, at a distance less than 1 mV or less than 10% of the distance between control value and the other endpoint, only the endpoint with the larger deviation was considered.

Table S1: **Table of the genetic variants used in this study.** The first column of the table shows the gene whose variant was studied in the named reference — see the caption of Table S2 to see which currents are affected by which gene. The second column shows the model parameters that are affected by the variant, "offm" and "offh" meaning the mid-points of activation and inactivation, respectively, "slom" and "sloh" their slopes, and "taum" and "tauh" their time constants. The variable "gamma" represents the factor by which ions included in inward $Ca^{2+}$ currents are added to the sub-membrane region, while the variable "Pup" represents the uptake rate of $Ca^{2+}$ ions by SERCA protein. The third column shows the direction and magnitude of the effect, ±x mV referring to a shift of the middle-point of the (in)activation curve by an absolute number of millivolts, and ±x% referring to a percentual change in the underlying quantity. In many cases, several variants were considered in a single study: Here, they are categorized by the type of the variant when necessary (e.g., in [S24] several variants of four loci, of which three were in pore-lining IS6 segment and two in bundle-crossing region of segment IIS6, were considered, and the variants are here categorized according to the segment they acted on). The fourth column names the type of variant used, while the fifth and sixth columns show the cell type in which the effects are measured and the animal species used in the study. The final column may give relevant extra information. The variants are listed in the same order as in Table 1. The table is an extended version of the corresponding table in [S25].

| Gene | Param. | Effect | Type of variant | Cell type | Animal | Notes |
|---|---|---|---|---|---|---|
| *CACNA1C* [S24] | offm<br>offh | -25.9..-1.4mV<br>-27.0..-3.8mV | L429T, L434T, S435T, S435A, S435P | TSA201 | human | These represent variant of the IS6 segment |
| *CACNA1C* [S24] | offm<br>offh | -37.3..-9.7mV<br>-30.0..-11.8mV | L779T, I781T, I781P | TSA201 | human | These are variants of IIS6 segment. Double (IIS6+IS6) mutant effects seem additive |
| *CACNA1C* [S26] | offm<br>slom<br>offh<br>sloh | -31.4..+7.0mV<br>-15..+45%<br>-28.5..+16.3mV<br>-28%..+38% | G432X, A780X, G1193X, A1503X | TSA201 | human / rabbit | Double mutant G432S/S435G not considered |
| *CACNA1C* [S27] | offm<br>slom | -38.5..+12.9mV<br>-54..+56% | I781X, C769P, G770P, N771P, I773P, F778P, L779P, A780P, A782P, V783P | TSA201 | human | Data for co-expression with both b1a and b2a. Here, the maximal deviation from the corresponding wild-type value taken |
| *CACNA1C* [S28] | offm<br>slom<br>offh | -27.8..+8.7mV<br>-11%..+14%<br>-19.1..+4.7mV | I781T, N785A, N785G, N785L | TSA201 | human | Double mutants not included (effects do not seem additive) |
| *CACNA1C* [S29] | offm<br>offh<br>sloh | -11.2..+1.0mV<br>-3.1..-0.3mV<br>+3%..+24% | Splice variants a1C77-A, -B, -C and -D | TSA201 | human | Slopes for activation curves visibly different, but fits not carried out (do not fit well to Boltzmann curve) |
| *CACNA1D* [S30] [S31] | offm<br>slom<br>offh<br>sloh<br>tauh | -10.9..-8.5mV<br>-27..-13%<br>-3.0..+3.5mV<br>-12..-19%<br>+25% | Splice variant 42A | TSA201 / HEK293 | human / rat / mouse | The splice variant is expressed in rat and human brain, thus both signs of effects could be possible. Here, the long form was considered the "control" and the short form (42A) the variant |
| *CACNA1D* [S30] [S31] | offm<br>slom<br>offh<br>sloh<br>tauh | -10.6..+3.4mV<br>-20..+12%<br>-5.3..+1.2mV<br>-34..-8%<br>-28% | Splice variant 43S | TSA201 / HEK293 | human / rat / mouse | The splice variant is expressed in rat and human brain, thus both signs of effects could be possible. Here, the long form was considered the "control" and the short form (43S) the variant |
| *CACNA1D* [S32] [S33] | offm<br>slom<br>tauh | +3.5..+6.6mV<br>-25..+19%<br>-50..+12% | Homozygous knock-out | AV node / chromaffin cells | mouse | Some time constants compared between single tau fits (as double tau fits not always well fitted) |
| *CACNA1D* [S34] | offm<br>slom<br>offh<br>sloh | -9.8mV<br>-20%<br>-15.4mV<br>+5% | A749G | TSA201 | human | Also G407R and co-transfection with WT studied, but in these cases the inactivation was too deficient to be included here |
| *CACNA1D* [S35] | offm<br>slom<br>offh<br>sloh<br>tauh | -24.2..+6.1mV<br>-30..+24%<br>-14.5..-3.6mV<br>-28%..+28%<br>+43%..+252% | V259D, I750M, P1336R | TSA201 | human | |
| *CACNA1D* [S36] | offm<br>slom<br>tauh | -17.8..-13.1mV<br>-19..-0%<br>-23%..+31% | rCav1.3scg variant and related mutants 7M2K, S244G, V1104A, and A2075V | TSA201 | human / rat | Double/triple mutants studied as well but they did not produce any stronger effects. Effects measured w.r.t. rCav1.3L |
| *CACNB2* [S37] | offh<br>sloh | -5.2mV<br>-31% | T11I | TSA201 | human | Small effects on offm ignored |
| *CACNB2* [S38] | taum | +70% | A1B2 vs A1 alone | HEK293 | human / mouse | |
| *CACNB2* [S39] | offm<br>offh<br>taum<br>tauh | -4.9..+4.9mV<br>-5.1..+5.1mV<br>-40%..+68%<br>-40%..+66% | Splice variants N1, N3, N4, N5 | HEK293 | human / mouse | |
| *CACNB2* [S40] | tauh | +26% | D601E | TSA201 | human | |
| *CACNA1I* [S41] | offm<br>offh<br>taum<br>tauh | -0.2..+1.3mV<br>-0.5..+1.6mV<br>-13..+45%<br>-20..+8% | Alternative splicing of exons 9 and 33 | HEK293 | human | Maximum of effects at -40 or 0 mV on kinetics considered. Changes in slopes were minuscule and thus ignored |
| *CACNA1I* [S42] | offm<br>slom<br>offh<br>sloh<br>taum<br>tauh | -4.3..-1.2mV<br>+5..+14%<br>-4.4..-1.9mV<br>-11%..+4%<br>-47%..-15%<br>-54%..+1% | Truncated cDNAs L4, L6 and L9 | HEK293 | human / rat | Changes in slopes and decays nonsignificant. Slopes included but time constants not (diverse asymptotes make comparison difficult) |
| *ATP2A2* [S43] [S44] | Pup<br>gamma | -34%<br>-30..-40% | Heterozygous null mutation | ESCs / myocytes | mouse | Here, the same variant is applied on both L5PC model (gamma changed) and SANC model (Pup changed) |
| *ATP2A2* [S45] | Pup | -78..+132% | G23E, S186F, C318R, P602L, D702N, P895L, S920Y(2a), S920Y(2b) | HEK293 | human | Some mutants had zero SERCA activity, these were ignored |
| *ATP2A2* [S46] | Pup | -80..-38% | N39D, N39T, DL41, DP42, C344Y, F487, K542X, G769R, Q790X, V843F, E917X, S920Y | HEK293 | human | Rates were estimated from figure. Comparison made against WT |
| *SCN1A* [S47] | offm<br>offh<br>slom<br>sloh | -0.3mV<br>+5.0mV<br>+15%<br>+23% | Q1489K | Cultured neocortical cells | human / rat | Slow inactivation could not be studied in detail in neurons |
| *SCN1A* [S48] | offm<br>offh<br>slom<br>sloh | +2.8mV<br>+6.3..+9.6mV<br>-1.6%<br>+4.2% | L1649Q | TSA201 | human | Electrophysiology done with the corresponding mutation L1636Q in the homologous SCN5A gene due to instabilities in recombinant bacteria |
| *SCN1A* [S49] | offm<br>offh<br>slom<br>sloh<br>tauh | -4.0mV<br>-5.8mV<br>-8%<br>+13%<br>+43..+47% | R859H | TSA201 | human | Persistent current also modified in mutant channels, but this is not taken into account here |
| *SCN1A* [S49] | offm<br>offh<br>slom<br>sloh<br>tauh | -8.1mV<br>+2.2mV<br>-3%<br>-3%<br>+26..+59% | R865G | TSA201 | human | Persistent current also modified in mutant channels, but this is not taken into account here |
| *SCN1A* [S50] | offm<br>slom<br>tauh | +6.0mV<br>+16%<br>+29% | T1174S | TSA201 | human | Data pooled with hbeta1 and hbeta2 subunit coexpression. Difference in inactivation nonsignificant |
| *SCN1A* [S51] | offm<br>slom<br>offh<br>sloh | +10.0mV<br>+15%<br>-0.6mV<br>+14% | M145T | TSA201 | human | |
| *HCN1* [S52] | offh<br>sloh | -2.1..-26.5mV<br>-12..-36% | D135W, D135H, D135N | HEK293 | mouse | Other mutations studied as well, but they changed the current too radically |
| *HCN1* [S53] | offh<br>sloh | -25.9..+17.7mV<br>-40..+3% | E229A, K230A, G231A, M232A, D233A, S234A, E235G, V236A, Y237A, EVY235-237DDD | Oocytes | mouse / xenopus | |
| *HCN1* [S54] | offh<br>tauh | +2.4..+3.9mV<br>-12..-0% | WAG-HCN1, WAG-HCN1 + HCN1 co-expression | Oocytes | rat / xenopus | Significance of effects not statistically tested |

We restricted our study to genes that have an important role on both SANCs and L5PCs and have been identified in SCZ GWASs, but the list is most probably incomplete. There are many $K^+$ channels that are important in both neurons and cardiac cells, e.g., the KCNQ family channels. These genes exceeded the maximal p-value threshold that we set ($3 \times 10^{-5}$), although some of them were close to the threshold (*KCNQ1*: $9.4 \times 10^{-5}$ and *KCNQ5*: $6.6 \times 10^{-5}$). Moreover, the function of KCNQ family ion channels has been proposed to be afflicted in SCZ patients through other mechanisms, such as phosphorylation [S55]. Another important set is the KCNN family of genes that code for subunits of $Ca^{2+}$-activated ion channels. These genes were excluded from the present study (although included in our earlier study [S25]), as their contribution to SANC activity is not included in the models we used. Nevertheless, they have been shown to be expressed in SANCs [S20] and therefore their variants could contribute to the neuronal-cardiac comorbidity symptoms as well. The presence of fast $Na^+$ channels in SANCs has been disputed in the past, but recent studies showed that they indeed are expressed [S56] and active [S19, 57] in SANCs, and thus they were included in both Kharche and Severi models.

## Scaling of gene variants

We ran simulations on all four cell models, separately exploring the effects of each variant of Table S1 on the cell behaviour. If the variant altered the cell behaviour dramatically, the genetic effect was scaled down (i.e., parameters were brought closer to the control values), so that there were no large discrepancies between the control cell and the mutant with the downscaled variant. This approach was used in order to simulate the SCZ-related SNP effects, many of which are known to be subtle (cf. [S58]) in contrast to the large phenotypic effects caused by the variants of Table 1.

To scale down the variants of Table S1 when implemented in the L5PC models, we followed the downscaling procedure as presented in [S25]. The downscaling criteria were the following:

**(A1)** Exactly 4 spikes should be induced as a response to somatic square-current injection of $A_1 \times 150$ ms,

**(A2)** Exactly 1 spike should be induced as a response to a distal (620 μm from soma) alpha-shaped synaptic conductance (time constant 5 ms, max. amplitude $A_2$),

**(A3)** Exactly 2 spikes should be induced as a response to a combined stimulus of somatic square-current injection ($A_{3a} \times$ 10 ms) and distal synaptic conductance (time constant 5 ms, max. amplitude $A_{3b}$, applied 5ms after the somatic pulse),

**(A4)** The integrated difference between the f-I curves of the considered neuron and the control neuron should not be more than 10% of the integral of the control neuron f-I curve, and

**(A5)** The membrane-potential limit cycle should not be too different from the control neuron limit cycle. In the Hay model, the neuron enters a tonic firing, and hence we set the condition A5 for the whole limit cycle set such that the difference $d_{cc}(lc_1, lc_2)$ should be $\leq 600$ (see the section below for details). In the Almog model, by contrast, the neuron enters a chattering type of firing with five spikes in each burst, and hence the full limit cycle consists of five sub-oscillations. To characterize the difference in the bursting kinetics, we chose to consider the phase-plane curves of the membrane potential between two successive spikes with the largest and smallest inter-spike interval — these intervals corresponded to the time from last spike of the previous burst to the first spike in the burst and the time from the first to the second spike. We set the criterion A5 such that both of these two phase-plane curves should be at a distance $d_{cc}(ppc_1, ppc_2) \leq 600$ from the corresponding curves in the control neuron.

The conditions A1–A3 restrict the magnitudes of short-time responses of the neuron, while the conditions A4–A5 concern continuous, steady-state firing. The above-mentioned amplitudes were chosen such that the control neuron most stably produces the named numbers of spikes with the default parameters — most stably in the sense that an equal change in current amplitude on logarithmic scale is required in order to produce one spike more or to produce one spike less. In the Hay model, these amplitudes were $A_1$ =0.696 nA, $A_2$ =0.0612 μS, $A_{3a}$ =1.137 nA, and $A_{3b}$ =0.100 μS. In the Almog model, these amplitudes were $A_1$ =0.781 nA, $A_2$ =0.0367 μS, $A_{3a}$ =0.699 nA, and $A_{3b}$ =0.0337 μS.

To scale down the variants of Table S1 when implemented in the SANC models, we defined the following criteria:

**(B1)** The difference between the pacemaking frequency in the considered SANC and the control SANC should not be more than 10% of control SANC frequency, and

**(B2)** The membrane-potential limit cycle should not be too different from the control neuron limit cycle ($d_{cc}(lc_1, lc_2) \leq B_1$, see the section below for details).

The constants $B_1$ were chosen as $B_1$=1500 in the Kharche model and $B_1$=500 in the Severi model. The larger constant for the Kharche model was chosen because relatively many variants produced $d_{cc}(lc_1, lc_2) \geq B_1$ while having a small effect (< 10%) on pacemaking frequency (86 out of 93 when $B_1$=500, compared to 64 out of 93 when $B_1$=1500), which was not the case in the Severi model (28 out of 93 for $B_1$=500).

In case one or more of the conditions A1–A5 or B1–B2 were violated when the cell with the considered variant was simulated, the effect was scaled down — all parameters in proportion — to a fraction $c < 1$ of the original effect where the violation is for the first time observed. These threshold effect parameters $c$ are listed in Table S2 for each variant together with the corresponding parameter changes, and the variants that were used in Figures 1–2 are highlighted. As we do not know how small a parameter change effect should be to correspond to a small SNP effect, we consider variants with different scalings where the threshold effect parameter $c$ is multiplied with another parameter $\epsilon < 1$. In this work, we consider the scaling parameter values $\epsilon = \frac{1}{2}$ and $\frac{1}{4}$, and we also display the effects of the corresponding opposite variants $\epsilon = -\frac{1}{2}$ and $-\frac{1}{4}$.

Table S2: **Table for the effects of the genetic variants on model parameters.** The first column names the gene and the study in which the gene variant was analyzed. The second column shows the effect of the variant on the model parameters, "offm" and "offh" meaning the mid-points of activation and inactivation, respectively, "slom" and "sloh" their slopes, and "taum" and "tauh" their time constants. The third, fourth, fifth, and sixth columns show the scaling parameters of the variant for all models. The rows separated by horizontal lines correspond to different entries of Table 1: If the corresponding study showed a large range of effects on single model parameters, the endpoints of such ranges are here treated as different variants that are downscaled independently of each other. The channel to which the changes are applied depends on the model as follows. In the Hay and Almog model, variants of *CACNA1C*, *CACNB2*, and *CACNA1D* affect the high-voltage activated $Ca^{2+}$ current $I_{CaHVA}$. Similarly, in the Severi model, these genes contribute to $I_{CaL}$. By contrast, the Kharche model makes a distinction between CaV1.2 and CaV1.3 L-type currents. Therefore, in the Kharche model, *CACNA1C* gene variants affect the current $I_{CaL,1.2}$, *CACNA1D* variants affect the current $I_{CaL,1.3}$, and *CACNB2* variants are in this work assumed to affect both $I_{CaL,1.2}$ and $I_{CaL,1.3}$ in the same magnitude. Variants of *CACNA1I* impact the low- or medium-voltage activated $Ca^{2+}$ currents ($I_{CaLVA}$ in the Hay model, $I_{CaMVA}$ in the Almog model, and $I_{CaT}$ in the Kharche and Severi model). In the Kharche and Severi models, the variants of *ATP2A2* affect the uptake rate of $Ca^{2+}$ into the sarcoplasmic reticulum (represented by a parameter named "Pup" in the Kharche and Severi models). The effects of these variants are not in general applicable to both SANC-type of models, where a model parameter describes the actual efficiency of the SERCA pump, and to L5PC-type of models, where a model parameter describes secondary effects on how large fraction of $Ca^{2+}$ finally enters the intracellular medium. An exception is a knock-out study by [S43], where the authors first studied the effects of a heterozygous null mutation on the SERCA activity in embryonic stem cells, and later analyzed the effects of the same operation on the intracellular $Ca^{2+}$ concentration in myocytes [S44]. The variants of *SCN1A* impact the fast sodium currents, named $I_{Nat}$ in the Hay and Almog model, $I_{Na}$ in the Severi model, and $I_{Na,1.1}$ in the Kharche model. The variants of *HCN1* impact the non-specific cation current, named $I_h$ in the L5PC models and $I_f$ in the SANC models. Asterisks (*) mark the variants used in Figures 1 and 2.

| Gene | Parameter changes | Hay | Almog | Kharche | Severi | |
|---|---|---|---|---|---|---|
| *CACNA1C* [S24] | offm: -25.9 mV, offh: -27 mV | $c = 0.066$ | $c = 0.078$ | $c > 2$ | $c = 0.102$ | * |
| *CACNA1C* [S24] | offm: -37.3 mV, offh: -30 mV | $c = 0.042$ | $c = 0.058$ | $c = 0.894$ | $c = 0.071$ | |
| *CACNA1C* [S26] | offm: -31.4 mV, slom: ×0.85, offh: -28.5 mV, sloh: ×0.72 | $c = 0.043$ | $c = 0.066$ | $c > 2$ | $c = 0.095$ | |
| | offm: +7 mV, slom: ×0.85, offh: -28.5 mV, sloh: ×0.72 | $c = 0.101$ | $c = 0.098$ | $c > 2$ | $c = 0.149$ | |
| | offm: -31.4 mV, slom: ×1.45, offh: -28.5 mV, sloh: ×0.72 | $c = 0.049$ | $c = 0.063$ | $c > 2$ | $c = 0.066$ | |
| | offm: +7 mV, slom: ×1.45, offh: -28.5 mV, sloh: ×0.72 | $c = 0.076$ | $c = 0.078$ | $c > 2$ | $c = 0.184$ | |
| | offm: -31.4 mV, slom: ×0.85, offh: +16.3 mV, sloh: ×0.72 | $c = 0.031$ | $c = 0.065$ | $c = 0.603$ | $c = 0.096$ | |
| | offm: +7 mV, slom: ×0.85, offh: +16.3 mV, sloh: ×0.72 | $c = 0.693$ | $c = 0.125$ | $c > 2$ | $c = 0.221$ | |
| | offm: -31.4 mV, slom: ×1.45, offh: +16.3 mV, sloh: ×0.72 | $c = 0.034$ | $c = 0.063$ | $c = 0.523$ | $c = 0.067$ | |
| | offm: +7 mV, slom: ×1.45, offh: +16.3 mV, sloh: ×0.72 | $c = 0.359$ | $c = 0.113$ | $c > 2$ | $c = 0.963$ | |
| | offm: -31.4 mV, slom: ×0.85, offh: -28.5 mV, sloh: ×1.38 | $c = 0.058$ | $c = 0.066$ | $c = 1.292$ | $c = 0.096$ | |
| | offm: +7 mV, slom: ×0.85, offh: -28.5 mV, sloh: ×1.38 | $c = 0.059$ | $c = 0.189$ | $c > 2$ | $c = 0.141$ | |
| | offm: -31.4 mV, slom: ×1.45, offh: -28.5 mV, sloh: ×1.38 | $c = 0.071$ | $c = 0.063$ | $c = 0.727$ | $c = 0.067$ | |
| | offm: +7 mV, slom: ×1.45, offh: -28.5 mV, sloh: ×1.38 | $c = 0.049$ | $c = 0.053$ | $c > 2$ | $c = 0.171$ | |
| | offm: -31.4 mV, slom: ×0.85, offh: +16.3 mV, sloh: ×1.38 | $c = 0.038$ | $c = 0.065$ | $c = 0.593$ | $c = 0.097$ | |
| | offm: +7 mV, slom: ×0.85, offh: +16.3 mV, sloh: ×1.38 | $c = 0.176$ | $c = 0.074$ | $c > 2$ | $c = 0.212$ | |
| | offm: -31.4 mV, slom: ×1.45, offh: +16.3 mV, sloh: ×1.38 | $c = 0.038$ | $c = 0.060$ | $c = 0.527$ | $c = 0.067$ | |
| | offm: +7 mV, slom: ×1.45, offh: +16.3 mV, sloh: ×1.38 | $c = 0.113$ | $c = 0.104$ | $c > 2$ | $c = 0.928$ | |
| *CACNA1C* [S27] | offm: -38.5 mV, slom: ×0.46 | $c = 0.028$ | $c = 0.050$ | $c = 0.603$ | $c = 0.096$ | |
| | offm: +12.9 mV, slom: ×0.46 | $c = 0.123$ | $c = 0.064$ | $c > 2$ | $c = 0.072$ | |
| *CACNA1C* [S28] | offm: -27.8 mV, slom: ×0.89, offh: -19.1 mV | $c = 0.052$ | $c = 0.054$ | $c = 1.200$ | $c = 0.105$ | |
| | offm: +8.7 mV, slom: ×0.89, offh: -19.1 mV | $c = 0.077$ | $c = 0.071$ | $c > 2$ | $c = 0.168$ | |
| | offm: -27.8 mV, slom: ×1.14, offh: -19.1 mV | $c = 0.057$ | $c = 0.073$ | $c = 0.919$ | $c = 0.086$ | |
| | offm: +8.7 mV, slom: ×1.14, offh: -19.1 mV | $c = 0.069$ | $c = 0.063$ | $c > 2$ | $c = 0.190$ | |
| | offm: -27.8 mV, slom: ×0.89, offh: +4.7 mV | $c = 0.042$ | $c = 0.074$ | $c = 0.713$ | $c = 0.106$ | |
| | offm: +8.7 mV, slom: ×0.89, offh: +4.7 mV | $c = 0.145$ | $c = 0.059$ | $c > 2$ | $c = 0.194$ | |
| | offm: -27.8 mV, slom: ×1.14, offh: +4.7 mV | $c = 0.044$ | $c = 0.062$ | $c = 0.664$ | $c = 0.086$ | |
| | offm: +8.7 mV, slom: ×1.14, offh: +4.7 mV | $c = 0.119$ | $c = 0.109$ | $c > 2$ | $c = 0.384$ | |
| *CACNA1C* [S29] | offm: -11.2 mV, offh: -3.1 mV, sloh: ×1.24 | $c = 0.157$ | $c = 0.171$ | $c > 2$ | $c = 0.239$ | |
| | offm: +1 mV, offh: -3.1 mV, sloh: ×1.24 | $c = 0.236$ | $c = 0.625$ | $c > 2$ | $c = 1.089$ | |
| *CACNA1D* [S30], [S31] | offm: -10.9 mV, slom: ×0.73, offh: -3 mV, sloh: ×0.81, tauh: ×1.25 | $c = 0.083$ | $c = 0.183$ | $c = 0.594$ | $c = 0.359$ | |
| | offm: -10.9 mV, slom: ×0.73, offh: +3.5 mV, sloh: ×0.81, tauh: ×1.25 | $c = 0.075$ | $c = 0.187$ | $c = 0.490$ | $c = 0.295$ | |
| *CACNA1D* [S30], [S31] | offm: -10.6 mV, slom: ×0.8, offh: -5.3 mV, sloh: ×0.66, tauh: ×0.72 | $c = 0.080$ | $c = 0.209$ | $c = 0.864$ | $c = 0.329$ | |
| | offm: +3.4 mV, slom: ×0.8, offh: -5.3 mV, sloh: ×0.66, tauh: ×0.72 | $c = 1.962$ | $c = 0.109$ | $c = 0.373$ | $c = 0.287$ | |
| | offm: -10.6 mV, slom: ×1.12, offh: -5.3 mV, sloh: ×0.66, tauh: ×0.72 | $c = 0.094$ | $c = 0.196$ | $c = 0.473$ | $c = 0.171$ | |
| | offm: +3.4 mV, slom: ×1.12, offh: -5.3 mV, sloh: ×0.66, tauh: ×0.72 | $c = 0.905$ | $c = 0.116$ | $c = 0.751$ | $c = 0.591$ | |
| | offm: -10.6 mV, slom: ×0.8, offh: +1.2 mV, sloh: ×0.66, tauh: ×0.72 | $c = 0.072$ | $c = 0.206$ | $c = 0.633$ | $c = 0.303$ | |
| | offm: +3.4 mV, slom: ×0.8, offh: +1.2 mV, sloh: ×0.66, tauh: ×0.72 | $c = 0.386$ | $c = 0.250$ | $c = 0.461$ | $c = 0.306$ | |
| | offm: -10.6 mV, slom: ×1.12, offh: +1.2 mV, sloh: ×0.66, tauh: ×0.72 | $c = 0.083$ | $c = 0.196$ | $c = 0.481$ | $c = 0.171$ | |
| | offm: +3.4 mV, slom: ×1.12, offh: +1.2 mV, sloh: ×0.66, tauh: ×0.72 | $c = 1.117$ | $c = 0.138$ | $c = 1.324$ | $c = 1.115$ | |
| *CACNA1D* [S32], [S33] | offm: +6.6 mV, slom: ×0.75, tauh: ×0.5 | $c = 0.190$ | $c = 0.047$ | $c = 0.248$ | $c = 0.192$ | |
| | offm: +6.6 mV, slom: ×1.19, tauh: ×0.5 | $c = 0.123$ | $c = 0.109$ | $c = 0.462$ | $c = 0.390$ | |
| | offm: +6.6 mV, slom: ×0.75, tauh: ×1.12 | $c = 0.209$ | $c = 0.104$ | $c = 0.418$ | $c = 0.163$ | |
| | offm: +6.6 mV, slom: ×1.19, tauh: ×1.12 | $c = 0.130$ | $c = 0.099$ | $c = 1.677$ | $c = 0.568$ | |
| *CACNA1D* [S34] | offm: -9.8 mV, slom: ×0.8, offh: -15.4 mV, sloh: ×1.05 | $c = 0.181$ | $c = 0.220$ | $c = 1.381$ | $c = 0.476$ | * |
| *CACNA1D* [S35] | offm: -24.2 mV, slom: ×0.7, offh: -14.5 mV, sloh: ×0.72, tauh: ×3.52 | $c = 0.045$ | $c = 0.083$ | $c = 0.211$ | $c = 0.178$ | |
| | offm: +6.1 mV, slom: ×0.7, offh: -14.5 mV, sloh: ×0.72, tauh: ×3.52 | $c = 0.318$ | $c = 0.125$ | $c = 0.528$ | $c = 0.115$ | |
| | offm: -24.2 mV, slom: ×1.24, offh: -14.5 mV, sloh: ×0.72, tauh: ×3.52 | $c = 0.053$ | $c = 0.083$ | $c = 0.183$ | $c = 0.133$ | |
| | offm: +6.1 mV, slom: ×1.24, offh: -14.5 mV, sloh: ×0.72, tauh: ×3.52 | $c = 0.152$ | $c = 0.041$ | $c = 1.369$ | $c = 0.287$ | |
| | offm: -24.2 mV, slom: ×0.7, offh: -14.5 mV, sloh: ×1.28, tauh: ×3.52 | $c = 0.059$ | $c = 0.078$ | $c = 0.222$ | $c = 0.178$ | |
| | offm: +6.1 mV, slom: ×0.7, offh: -14.5 mV, sloh: ×1.28, tauh: ×3.52 | $c = 0.105$ | $c = 0.093$ | $c = 0.510$ | $c = 0.113$ | |
| | offm: -24.2 mV, slom: ×1.24, offh: -14.5 mV, sloh: ×1.28, tauh: ×3.52 | $c = 0.074$ | $c = 0.080$ | $c = 0.191$ | $c = 0.135$ | |
| | offm: +6.1 mV, slom: ×1.24, offh: -14.5 mV, sloh: ×1.28, tauh: ×3.52 | $c = 0.076$ | $c = 0.172$ | $c = 1.277$ | $c = 0.260$ | |
| *CACNA1D* [S36] | offm: -17.8 mV, slom: ×0.81, tauh: ×0.77 | $c = 0.065$ | $c = 0.118$ | $c = 0.359$ | $c = 0.178$ | |
| | offm: -17.8 mV, slom: ×0.81, tauh: ×1.31 | $c = 0.063$ | $c = 0.116$ | $c = 0.311$ | $c = 0.211$ | |
| *CACNB2* [S37] | offh: -5.2 mV, sloh: ×0.69 | $c = 0.381$ | $c = 1.116$ | $c = 1.909$ | $c = 1.498$ | |
| *CACNB2* [S38] | tauh: ×1.7 | $c > 2$ | $c = 1.689$ | $c = 1.484$ | $c = 0.782$ | |
| *CACNB2* [S39] | offm: -4.9 mV, offh: -5.1 mV, taum: ×0.6, tauh: ×0.6 | $c = 0.194$ | $c = 0.437$ | $c = 0.847$ | $c = 0.321$ | |
| | offm: +4.9 mV, offh: -5.1 mV, taum: ×0.6, tauh: ×0.6 | $c = 0.278$ | $c = 0.120$ | $c = 0.422$ | $c = 0.377$ | |
| | offm: -4.9 mV, offh: +5.1 mV, taum: ×0.6, tauh: ×0.6 | $c = 0.131$ | $c = 0.467$ | $c = 0.581$ | $c = 0.320$ | |
| | offm: +4.9 mV, offh: +5.1 mV, taum: ×0.6, tauh: ×0.6 | $c = 1.101$ | $c = 0.145$ | $c = 0.712$ | $c = 0.610$ | |
| | offm: -4.9 mV, offh: -5.1 mV, taum: ×1.68, tauh: ×0.6 | $c = 0.814$ | $c = 0.438$ | $c = 0.935$ | $c = 0.348$ | |
| | offm: +4.9 mV, offh: -5.1 mV, taum: ×1.68, tauh: ×0.6 | $c = 0.105$ | $c = 0.146$ | $c = 0.316$ | $c = 0.310$ | |
| | offm: -4.9 mV, offh: +5.1 mV, taum: ×1.68, tauh: ×0.6 | $c = 0.373$ | $c = 0.406$ | $c = 1.016$ | $c = 0.348$ | |
| | offm: +4.9 mV, offh: +5.1 mV, taum: ×1.68, tauh: ×0.6 | $c = 0.144$ | $c = 0.118$ | $c = 0.445$ | $c = 0.499$ | |
| | offm: -4.9 mV, offh: -5.1 mV, taum: ×0.6, tauh: ×1.66 | $c = 0.189$ | $c = 0.434$ | $c = 0.469$ | $c = 0.648$ | |
| | offm: +4.9 mV, offh: -5.1 mV, taum: ×0.6, tauh: ×1.66 | $c = 0.342$ | $c = 0.127$ | $c = 1.657$ | $c = 0.319$ | |
| | offm: -4.9 mV, offh: +5.1 mV, taum: ×0.6, tauh: ×1.66 | $c = 0.122$ | $c = 0.398$ | $c = 0.363$ | $c = 0.506$ | |
| | offm: +4.9 mV, offh: +5.1 mV, taum: ×0.6, tauh: ×1.66 | $c = 1.687$ | $c = 0.188$ | $c > 2$ | $c = 0.338$ | |
| | offm: -4.9 mV, offh: -5.1 mV, taum: ×1.68, tauh: ×1.66 | $c = 0.707$ | $c = 0.395$ | $c = 1.984$ | $c = 0.729$ | |
| | offm: +4.9 mV, offh: -5.1 mV, taum: ×1.68, tauh: ×1.66 | $c = 0.113$ | $c = 0.188$ | $c = 0.960$ | $c = 0.298$ | * |
| | offm: -4.9 mV, offh: +5.1 mV, taum: ×1.68, tauh: ×1.66 | $c = 0.320$ | $c = 0.383$ | $c = 1.128$ | $c = 0.838$ | |
| | offm: +4.9 mV, offh: +5.1 mV, taum: ×1.68, tauh: ×1.66 | $c = 0.157$ | $c = 0.254$ | $c = 1.426$ | $c = 0.313$ | |

Table 2 continued.

| Gene | | Parameter changes | Hay | Almog | Kharche | Severi | |
|---|---|---|---|---|---|---|---|
| *CACNB2* | [S40] | tauh: ×1.26 | $c > 2$ | $c > 2$ | $c > 2$ | $c = 1.796$ | |
| *CACNA1I* | [S41] | offm: +1.3 mV, offh: +1.6 mV, taum: ×0.87, tauh: ×0.8 | $c > 2$ | $c = 1.764$ | $c > 2$ | $c > 2$ | |
| | | offm: +1.3 mV, offh: +1.6 mV, taum: ×1.45, tauh: ×0.8 | $c > 2$ | $c = 0.187$ | $c > 2$ | $c > 2$ | * |
| *CACNA1I* | [S42] | offm: -4.3 mV,slom: ×1.14,offh: -4.4 mV,sloh: ×0.89,taum: ×0.53,tauh: ×0.46 | $c = 0.968$ | $c = 0.290$ | $c = 0.580$ | $c > 2$ | |
| | | offm: -4.3 mV,slom: ×1.14,offh: -4.4 mV,sloh: ×1.04,taum: ×0.53,tauh: ×0.46 | $c > 2$ | $c = 0.233$ | $c = 0.711$ | $c > 2$ | |
| *ATP2A2* | [S43], [S44] | Pup: ×0.66 or gamma: ×0.6 | $c = 0.179$ | $c = 0.188$ | $c > 2$ | $c > 2$ | * |
| *ATP2A2* | [S45] | Pup: ×0.22 | | | $c = 1.527$ | $c = 1.841$ | |
| | | Pup: ×2.31 | | | $c > 2$ | $c = 0.541$ | |
| *ATP2A2* | [S46] | Pup: ×0.2 | | | $c = 1.436$ | $c = 1.735$ | |
| *SCN1A* | [S47] | offm: -0.3 mV, offh: +5 mV, slom: ×1.15, sloh: ×1.23 | $c = 0.049$ | $c = 0.005$ | $c > 2$ | $c = 0.778$ | * |
| *SCN1A* | [S48] | offm: +2.8 mV, offh: +9.6 mV, slom: ×0.984, sloh: ×1.042 | $c = 0.063$ | $c = 0.015$ | $c > 2$ | $c = 0.930$ | |
| *SCN1A* | [S49] | offm: -4 mV, offh: -5.8 mV, slom: ×0.92, sloh: ×1.13, tauh: ×1.47 | $c = 0.273$ | $c = 0.223$ | $c > 2$ | $c > 2$ | |
| *SCN1A* | [S49] | offm: -8.1 mV, offh: +2.2 mV, slom: ×0.97, sloh: ×0.97, tauh: ×1.59 | $c = 0.037$ | $c = 0.004$ | $c = 1.438$ | $c = 1.038$ | |
| *SCN1A* | [S50] | offm: +6 mV, slom: ×1.16, tauh: ×1.29 | $c = 0.129$ | $c = 0.005$ | $c > 2$ | $c > 2$ | * |
| *SCN1A* | [S51] | offm: +10 mV, offh: -0.6 mV, slom: ×1.15, sloh: ×1.14 | $c = 0.062$ | $c = 0.003$ | $c > 2$ | $c > 2$ | |
| *HCN1* | [S52] | offh: -26.5 mV, sloh: ×0.64 | $c = 0.296$ | $c = 0.008$ | $c = 0.614$ | $c = 0.145$ | * |
| *HCN1* | [S53] | offh: -25.9 mV, sloh: ×0.6 | $c = 0.282$ | $c = 0.007$ | $c = 0.580$ | $c = 0.147$ | |
| | | offh: +17.7 mV, sloh: ×0.6 | $c = 0.807$ | $c = 0.092$ | $c > 2$ | $c = 0.283$ | |
| *HCN1* | [S54] | offh: +3.9 mV, tauh: ×0.88 | $c = 1.226$ | $c = 0.101$ | $c > 2$ | $c = 0.872$ | * |

For those unscaled variants that did not violate the conditions A1–A5 or B1–B2, we sought for the threshold effect up to twice the original effect, i.e., $c < 2$. If the variant still obeyed the conditions, we considered the original variant as the $\epsilon = \frac{1}{2}$ variant and applied other scalings with respect to this choice.

The model parameters affected by the variants include quantities of various roles and dimensions (mV, mM, ms, etc.), which calls for a careful consideration how to scale them properly. We chose to perform this such that those parameters that may receive both negative and positive values were scaled linearly, while the parameters that receive only positive values were scaled on the logarithmic scale. In practice, this means that the differences in offset and reverse potentials ($V_{\mathrm{offm}*}$, $V_{\mathrm{offh}*}$, $E_{\mathrm{Ih}}$) between control and variant neuron were expressed as an additive term ($\pm x$ mV), and this term $x$ was multiplied by a parameter $c \in [0, 1]$ in the downscaling procedure. By contrast, the differences in all the other model parameters ($V_{\mathrm{offc}*}$, $V_{\mathrm{slo}*}$, $\tau_*$, $c_*$, $\gamma_*$) between control and variant neuron were expressed as a multiplication ($\times x$), where the downscaling caused this factor $x$ to be exponentiated by the same parameter $c$. This procedure permits a continuum of parameter changes in the range $c \in [0, 1]$ that is directly applicable to amplified ($c > 1$) parameter changes as well.

Most of the variants of Table S2 affect many model parameters at once, but some of them — typically the offset potentials — may have a larger role in determining the magnitude of the scaling parameters. Figure S1 illustrates the distribution of the effects of downscaled *SCN1A* variants on pairs of model parameters and how they relate to the boundary conditions implied solely by the two model parameters in the Hay model. In Figure S1A, the variant effects on offset potentials of activation and inactivation of transient $Na^+$ channels are shown, and in Figure S1B, the effects on voltage-sensitivites of activation and inactivation of transient $Na^+$ channels are shown. The areas inside the scaling perimeters represent the sets of the pairs of considered model parameters that do not violate any of the scaling conditions. Hence, if a variant only affects the two model parameters, the $\epsilon = 1$ version of this variant (red symbols) should lie on the perimeter, and the $\epsilon = \frac{1}{2}$ and $\epsilon = \frac{1}{4}$ versions between this point and the origin. However, all of the *SCN1A* variants impact both offset potential (of either activation or inactivation or both) and voltage-sensitivity (slope), and hence this is never the case. The variants where the $\epsilon = 1$ version is located outside the scaling perimeter must have a compensatory effect of other impacted model parameters, while in the variants where the $\epsilon = 1$ version is inside the scaling perimeter, the other impacted model parameters strengthen the effect of the shown model parameters, and hence the scaling conditions are violated sooner (than in the case where only the two shown model parameters where impacted, i.e., the scaling perimeter).

The scaling perimeter of Figure S1A is skewed such that it allows larger change in the offset parameter of activation if there is an analogous change in the offset parameter of inactivation, and vice versa. If the changes are non-analogous (e.g., offset of activation moved toward positive potentials but offset of inactivation moved toward negative potentials), the scaling condition A5 (coherency of the limit cycle) is violated. If both offsets are moved toward positive potentials, the effects on limit cycle are constrained, but the scaling condition A2 is violated (no spikes as a response to an EPSP-like stimulus), whereas if both offsets are moved toward negative potentials, the scaling condition A3 is violated (an extra spike emitted as a response to a combination of somatic and apical EPSP-like stimulus). The scaling perimeter of Figure S1B, in turn, is skewed such that it allows larger change in the slope parameter of activation if there is an opposite change in the slope parameter of inactivation. This is intuitive, as the inactivation curve mid-point is at more hyperpolarized potential than that of the activation curve: When the activation slope is made steeper, the channels are activated at more depolarized potentials, and to allow larger values of inactivation curve at more depolarized potentials, the inactivation slope has to be less steep. Instead of scaling conditions A2 and A3, which were violated in Figure S1A, in Figure S1B the condition A4 (f-I curve coherency) is violated at the thinner ends of the scaling perimeter.

Our framework allows studying SNP-like variants with small effects by using downscaled data from genetic variants of larger phenotypic effects, and is therefore dependent on how this downscaling is performed. Here, following the framework of [S25], we introduced scaling conditions (A1–A5 and B1–B2) that restrict the effects of the variants on cellular phenotypes (thresholds of firing, spiking and pacemaking frequencies, and membrane-potential limit cycles). In a similar fashion to [S25], we required that the variant neuron respond with the equal number of spikes to short stimuli at soma and apical dendrite as the control neuron (conditions A1–A3). Moreover, we required that the firing and pacemaking rates differ by less than 10% with respect to the control cell firing/pacemaking rate. The real effect sizes in common SCZ-associated SNPs may well be smaller than the hand-picked effect size, whereas we consider it unlikely that they be larger than this due to the large number of associated SNPs. As an example, in [S59], complete knock-kout of *CACNA1D* or *CACNA1G* gene caused arrhythmia in mice and decreased the SAN pacemaking rate by approximately 50% or 30%, respectively, and thus represent life-threatening phenotypes [S59]. Combinations of variants that alter the frequency of action potentials by more than 10% (especially if they all decrease the intrinsic excitability) could easily render the cell functionality to a pathological regime such as the ones found experimentally by complete knock-outs. This is especially true for cardiac pacemaking cells — see the devastating effects of only seven $\epsilon = \frac{1}{2}$ variants obeying the 10%-rule (condition B1) in Fig. S10.

## A distance metric for membrane-potential phase-plane curves

The distance metric for limit cycles, as defined in [S25], can be used as a distance metric for phase-plane curves in general. A membrane-potential phase-plane curve can be described as a 1-dimensional manifold in the space $\mathcal{LC} = \mathcal{V} \times d\mathcal{V}$, where $\mathcal{V}$ is the space of possible values for membrane potentials and $d\mathcal{V}$ is the space of possible values for time derivatives of membrane potential. Due to the difference in units for $x$ and $y$ axis, there is no obvious metric for this space, but such can easily be constructed. We define a bijection (an invertible function) $f : \mathcal{LC} \to \mathbb{R}^2$ as

$$f(V, dV) = \left( \frac{V}{1.0 \text{ mV}}, \frac{dV}{C} \right),$$

where the constant $C$ was chosen such that the control-cell limit cycle spans an equal range on $x$ and $y$ axes when the soma is given a DC of amplitude 1.0 nA. This constant was $C$ =7.029 mV/ms in the Hay model (for a somatic DC input of 1.0 nA), $C$ =4.293 mV/ms in the Almog model (for a somatic DC input of 0.8 nA), $C$ =7.762 mV/ms in the Kharche model, and $C$ =9.300 mV/ms in the Severi model. Now, a distance metric in $d : \mathcal{LC} \times \mathcal{LC}$ can be defined as

$$d(x, y) = ||f(x) - f(y)||_2,$$

where $|| \cdot ||_2$ is a Euclidean norm, i.e. mean square error. Furthermore, the distance of a point $x \in \mathcal{LC}$ from a phase-plane curve $lc \subset \mathcal{LC}$ can be defined as

$$d_c(x, lc) = \min_{y \in lc} ||f(x) - f(y)||.$$

To evaluate the difference between two phase-plane curves $lc_1, lc_2 \subset \mathcal{LC}$, we define

$$d_{cc}(lc_1, lc_2) = \frac{1}{2} \int_{lc_1} d_c(x, lc_2)dx + \frac{1}{2} \int_{lc_2} d_c(x, lc_1)dx.$$

Using the average of the two integrals assures that no part of either phase-plane curve is ignored: If only the integral over points of $lc_1$ was used, the phase-plane curve $lc_2$ could have any "extra" part, for example an unusually long and slow hyperpolarization period or minor sub-oscillations, that might largely be disregarded in the integration as there are always some other points in $lc_2$ that lie nearer to $lc_1$.

# Supplemental results — single cell

## Single-parameter analysis

Figures 1, 2 and 3 showed that most of the $Na^+$ and HCN channel variants had largely similar effects on L5PC and SANC excitability, while $Ca^{2+}$ channel and $Ca^{2+}$ transporter variants had mostly opposite effects. Most of the underlying variants (see Table S2) have simultaneous effects on many model parameters, and in certain cases, these parameter changes may cancel out each other's effects. Here, we show results for artificial variants, where only one model parameter is changed at a time. Figure S2 shows the effects of lowered threshold for activation and inactivation of $Na^+$ channels and inactivation of HCN channels, while Figure S3 shows the effects of lowered threshold for activation and inactivation of $Ca^{2+}$ channels. Figure S4 shows the single-parameter effects of all parameters that were varied in Figure 3, and Table S3 shows the correlations of these data in a similar manner as shown for variants of Figure 3 in Table 2.

## Variants with inconsistent effects in the two models of the same cell type

The *CACNA1I* variant shown in Figure 2 is an exception to the trend shown by the rest of the variants in Figures 2 and 3, as in the Hay model it has a minuscule effect and in the Severi model a small, yet opposite effect to that of the Kharche model. This conflict is not unexpected as the considered variant has very similar effects on the voltage-dependencies of activation and inactivation — both mid-points are increased and both dynamics are made faster — and hence the effects on cell behavior are difficult to predict *a priori*. As a similar example, consider the second-to-last variant (unscaled) of Table S2, which increases the offset potential by 17.7 mV of the $I_h$ current and steepens the voltage-sensitivity slope by 40%. This variant has qualitatively similar effect on the $I_h$ channel in the two L5PC models in strongly hyper-polarized potentials (gain of function, due to steeper slope) and strongly depolarized potentials (loss of function, due to steeper slope). However, the change from gain-of-function to loss-of-function variant occurs at different potentials in the two models: In the Hay model, the variant is loss-of-function from -73 mV upwards, while in the Almog model, the variant is loss-of-function only from -3 mV upwards (data not shown). This is reflected in the firing properties in Figure 3, where the (downscaled) variant shows decreased firing rate in the Hay model but increased firing in the Almog model. The same variant caused qualitatively different effects on the pacemaking frequency in the two SANC models as well.

Due to the different ion-channel kinetics in the two L5PC models, even simple (single-parameter) variants may have qualitatively different or in other ways inconsistent effects on the firing rates. In Figures S5 and S6, the effect of lowered threshold for activation of transient $Na^+$ channels in the Hay and Almog models, respectively, is analyzed in more detail. The variant effects on the time courses of each modeled current (averaged over the whole membrane) are shown, and the origin of increased or decreased firing rate can be observed. In the Hay model, the immediate effect of lowered threshold for $Na^+$ channel activation is an increase of the $Na^+$ current amplitude during spike (third row in Figure S5B and S5D, purple curves). This has downstream effects on other currents, especially, the amplitudes of HVA and LVA $Ca^{2+}$ currents, which are also increased during spikes (fifth and sixth rows in Figure S5B and S5D). The increased $Ca^{2+}$ currents in turn cause an elevation in the $Ca^{2+}$ concentration and thus an increased amplitude of the SK current. This effect lasts during the inter-spike interval (Figure S5C), and is the key in causing the delay for the next spike, despite the depolarizing effect of the lowered activation potential of transient $Na^+$ channels. Other currents ($I_{Nap}$, $I_h$, and $I_{Kv3.1}$) have opposing effects due to the altered membrane potential, as seen in Figure S5C, but the change in their amplitude is in this example smaller than that in the SK currents.

Figure S6 illustrates the effects of the same single-parameter variant as in S5, but in the Almog model. Due to the bursting behavior, the time courses are divided using a finer division than in S6, and an additional panel (Figure S6E) is used to capture the difference between variants during the inter-burst intervals. In the Almog model, the amplitudes of the

currents are nearly unchanged during spikes in a variant with a lowered threshold for $Na^+$ channel activation, as seen in Figure S6C (purple lines). However, during the inter-burst interval, the $Na^+$ currents are markedly increased, as shown by the curves of Figure S6D and the temporal integration of these data in Figure S6E. This causes the neuron to fire (and start the following burst) ahead of time in comparison to the control neuron, as shown in Figure S6F. The hastened firing has many downstream effects, most important of which is seen in a slightly increased $Ca^{2+}$ concentration amplitude (second row in Figure S6C and S6F) and the hence increased SK and BK currents (seventh and eighth rows in Figure S6D and S6E), but their effect is minor compared to the change in $Na^+$ current (Figure S6E). These observations are in line with the data corresponding to this variant in Figure S4, where the effect of the variant is dubious in the Hay model and clearly excitatory in the Almog model.

In a similar fashion, Figures S7 and S8 show the analysis on the effects of lowered threshold for activation of HVA $Ca^{2+}$ channels in the Hay and Almog models, respectively. In both models, the variant shows an increased HVA $Ca^{2+}$ current both during (fifth row in Figure S7B and fourth row in S8B-C) and after the firing (fifth row in Figure S7C and fourth row in S8D-E). This increases the $Ca^{2+}$ concentration, which causes an increase in the SK current, and the increased SK current delays the induction of the next spike in comparison to the control neuron (Figure S7D and S8F). Due to the altered membrane-potential time courses, there are opposite changes in other current species during the inter-spike (Hay model, Figure S7C) or inter-burst (Almog model, S8D-E) interval. Most notable of these changes are the decreased $I_{Kv3.1}$ amplitude in the Hay model, the decreased $I_{Kt}$ amplitude in the Almog model, and increased $I_h$ amplitude in both models. The summed effect of these changes is, nevertheless, smaller than the SK-mediated delaying effect on the following spike.

## Combinations of variants may cause large effects

A likely scenario in a polygenic disorder such as SCZ is that the combined effects of alterations in different genetic mechanisms raise the risk of observing disease phenotypes, although the effect of each of the alterations alone is small. In this section, we illustrate the range of effects that such combinations can have on both L5PC firing and SANC pacemaking. Basing on the results of Figure 3, we chose for the seven considered genes the variants that produced the maximal or minimal firing rate in the Hay or Almog-model neuron. The selected variants are marked with 'x's and 'o's in Figure 3. We then simulated the L5PC firing and SANC pacemaking using the combination of the parameter changes corresponding to these variants. The results are shown in Figure S10.

Figure S10 shows that combinations of variants may radically change the L5PC and SANC behavior. The combinations of those $\epsilon = \frac{1}{2}$ variants that produced the maximal L5PC firing rates made the Kharche-model cells cease pacemaking. By contrast, the combinations of $\epsilon = \frac{1}{2}$ variants that produced the minimal firing rates in the L5PC models did not have a large effect. In general, the variant combinations that increase the firing rate in L5PCs decrease the pacemaking rate in SANCs and vice versa. This is expected, as the majority (4–5 out of 6–7) of the variants in the combinations were variants of genes encoding $Ca^{2+}$ channel or transporters.

# Supplemental results — signal propagation

## Changes in SANC excitability affect the signal propagation in a chain of SANCs

We implemented a simple model of interconnected SANCs to analyze the effects of the variants on signal propagation rate. We simulated a chain of 200 Kharche-model SANCs, where the distance of two neighboring cells was set to 5 μm and the membrane potentials were coupled with a diffusion constant of $6\times10^{-5}$ $m^2/s$ [S60]. In these simulations, we first applied a hyperpolarizing voltage clamp to the valley potential of the control SANC oscillation (-64 mV) for a 1000-ms duration. After this clamp, 10% of the cells (located at one end of the chain) were applied a depolarizing voltage clamp to the peak potential of SANC oscillation (23 mV), while the remaining 90% were released. This created a depolarizing wave that proceeded from the first 20 SANCs throughout the chain as an interplay of passive signal propagation (restricted by the diffusion constant) and the activation and inactivation of the ion channels involved in the pacemaking. The delay of the conduction of this wave can be considered a correlate of the signal propagation delay in the sinoatrial node (SAN), although many details are missing from this analysis — see e.g. [S60] for the difference between central (the initiation zone of the cardiac action potential) and peripheral (activated with a delay with respect to the central zone) SANCs. We quantified the conduction delay both in control and variant SANCs, and the results for several $Ca^{2+}$ channel and transporter variants are shown in Figure S9. Figure S11 shows for all variants the time at which the end node (at 1 mm) reaches 20 mV membrane potential.

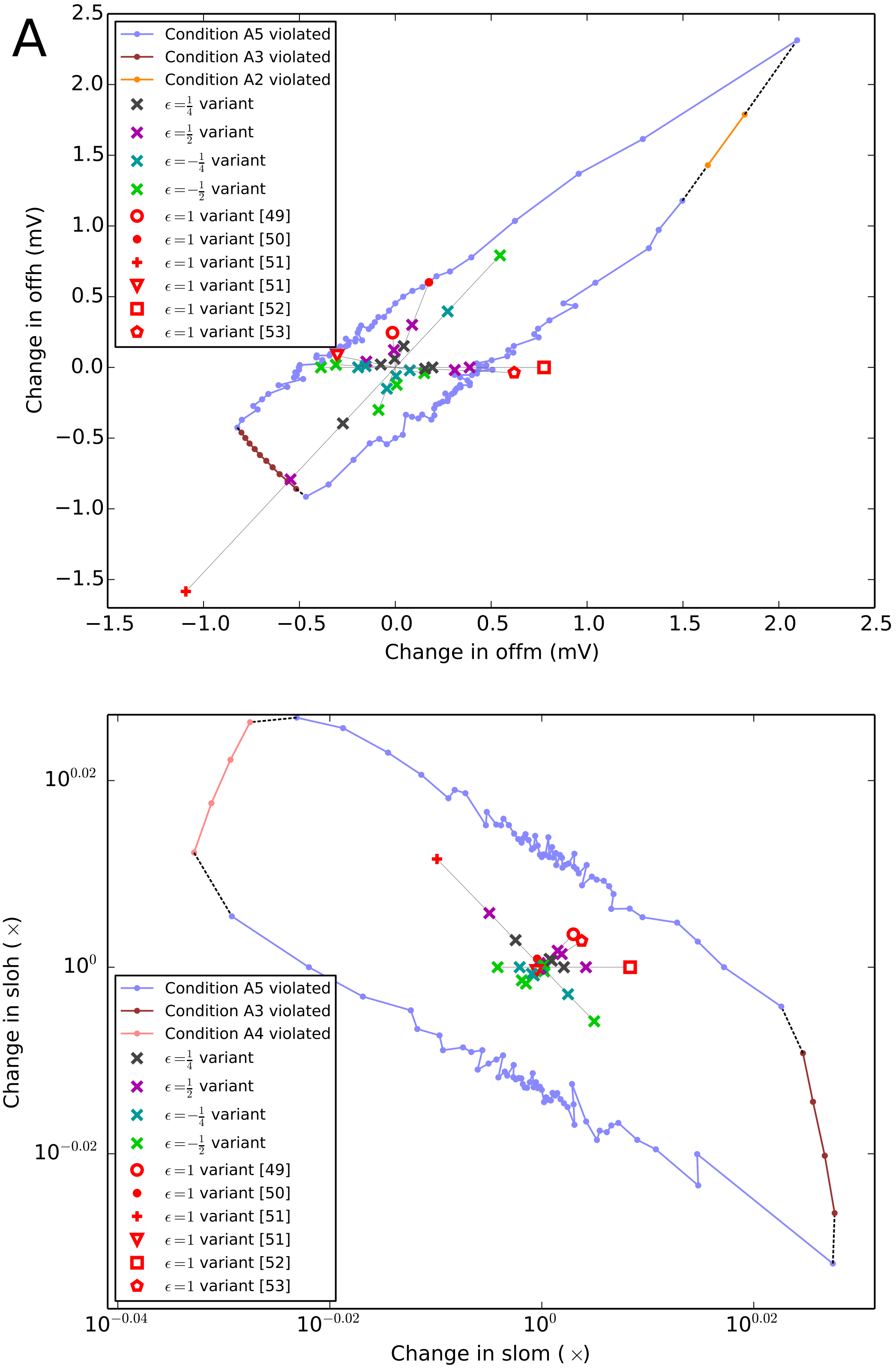


Figure S1: **Effects of downscaled *SCN1A* variants on channel activation and inactivation parameters.** The red symbols ($\epsilon = 1$) and magenta ($\epsilon = \frac{1}{2}$), gray ($\epsilon = \frac{1}{4}$), cyan ($\epsilon = -\frac{1}{4}$), and green ($\epsilon = -\frac{1}{2}$) crosses represent different versions of the considered variants (see Table S2). **A**: The x axis shows the variant effect on the offset potential of activation, and the y axis shows the effect on the offset potential of inactivation. **B**: The x axis shows the variant effect on the voltage-sensitivity of the activation curve, and the y axis shows the effect on the voltage-sensitivity of the inactivation curve (the respective model parameters are multiplied by the values shown by the axes). The perimeters around origins show the scaling parameters of artificial variants, where only these two model parameters are varied and others are kept fixed. The perimeters are formed by 120 points, each independently scaled, and the color of the point on the perimeter represents the scaling condition which is first violated when crossing the perimeter. The variants show a scattered distribution across the planes, which confirms that the variants span a large array of altered ion-channel dynamics.

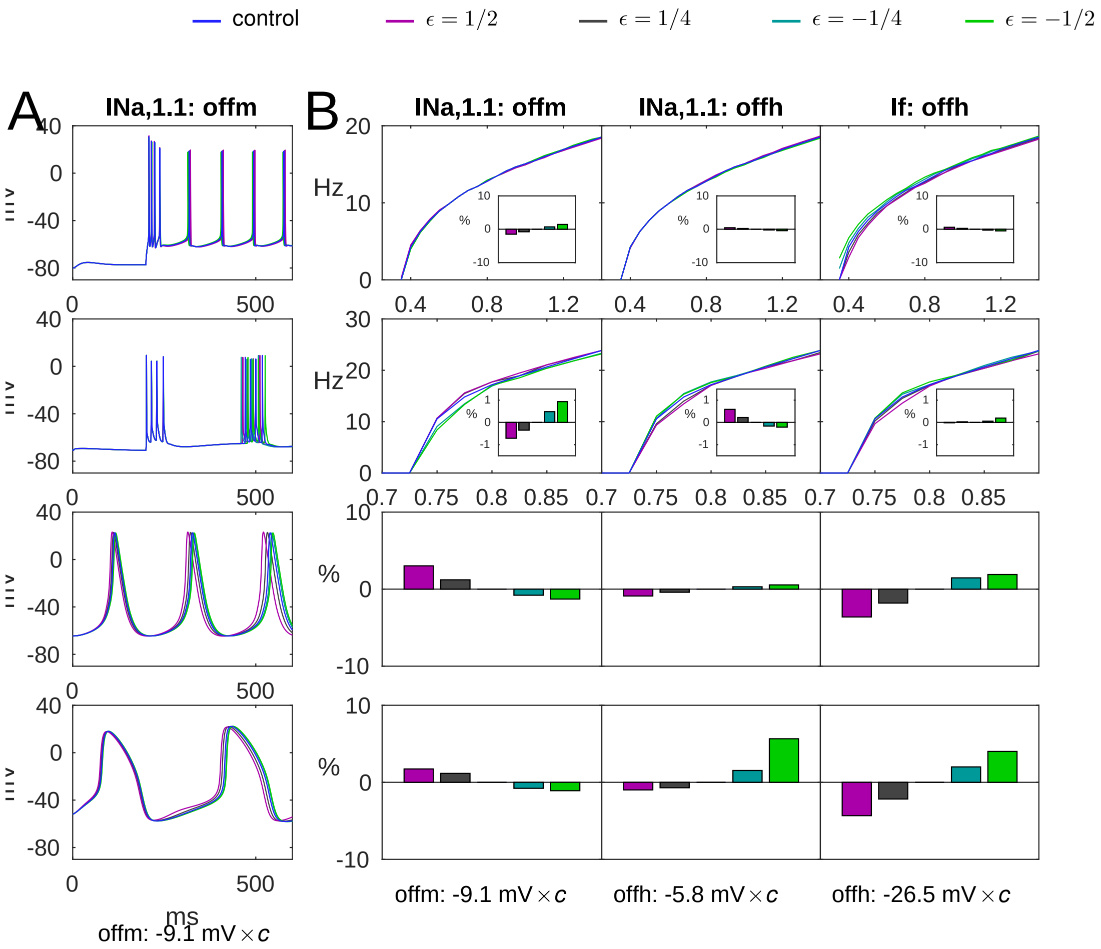


Figure S2: **Effects of $Na^+$ and HCN channel (in)activation thresholds on L5PC firing and SANC pacemaking are qualitatively similar.** **A**: The membrane-potential time courses of the modeled cells in control cells and $Na^+$ channel variant cells. The L5PCs are stimulated with a somatic DC, while the SANCs rhythmically fire cardiac action potentials without any external stimulus. **B**: The f-I curves and pacemaking rhythms of the modeled cells for different $Na^+$ channel and HCN channel variants. The insets for the L5PC model results show the change in threshold current at rest in relation to that of the control neuron. For SANCs, the relative difference from control neuron pacemaking rhythm are shown. The models from top to bottom: Hay, Almog, Kharche, and Severi. Different colors represent different scalings of the same variant (blue: control, magenta: $\epsilon = \frac{1}{2}$, gray: $\epsilon = \frac{1}{4}$, cyan: $\epsilon = -\frac{1}{4}$, green: $\epsilon = -\frac{1}{2}$). See Figure 1 for details.

Table S3: **Firing frequencies of L5PCs and pacemaking frequencies of SANCs show a correlation for single-parameter variants of $Na^+$ and HCN channel, and a subtle anti-correlation for single-parameter variants of $Ca^{2+}$ channels and transporters.** The table shows the correlation coefficients between the firing or pacemaking frequencies of $\epsilon = \frac{1}{2}$ variants (see Figure S4), as predicted by the different models. Left: Only data from parameters governing $Na^+$ and non-specific ion currents included. Middle: Only data from parameters governing $Ca^{2+}$ currents and intracellular $Ca^{2+}$ dynamics included. Right: All parameters included.

$Na^+$ and HCN channel variants

| | Hay | Almog |
|---|---|---|
| Kharche | 0.5136 | 0.7473 |
| Severi | 0.2726 | 0.7489 |

$Ca^{2+}$ channel and transporter variants

| | Hay | Almog |
|---|---|---|
| Kharche | -0.2221 | -0.5704 |
| Severi | -0.1615 | -0.3518 |

All variants

| | Hay | Almog | Kharche | Severi |
|---|---|---|---|---|
| Hay | 1 | 0.3828 | -0.1304 | -0.0981 |
| Almog | 0.3828 | 1 | -0.2119 | -0.0489 |
| Kharche | -0.1304 | -0.2119 | 1 | 0.5853 |
| Severi | -0.0981 | -0.0489 | 0.5853 | 1 |

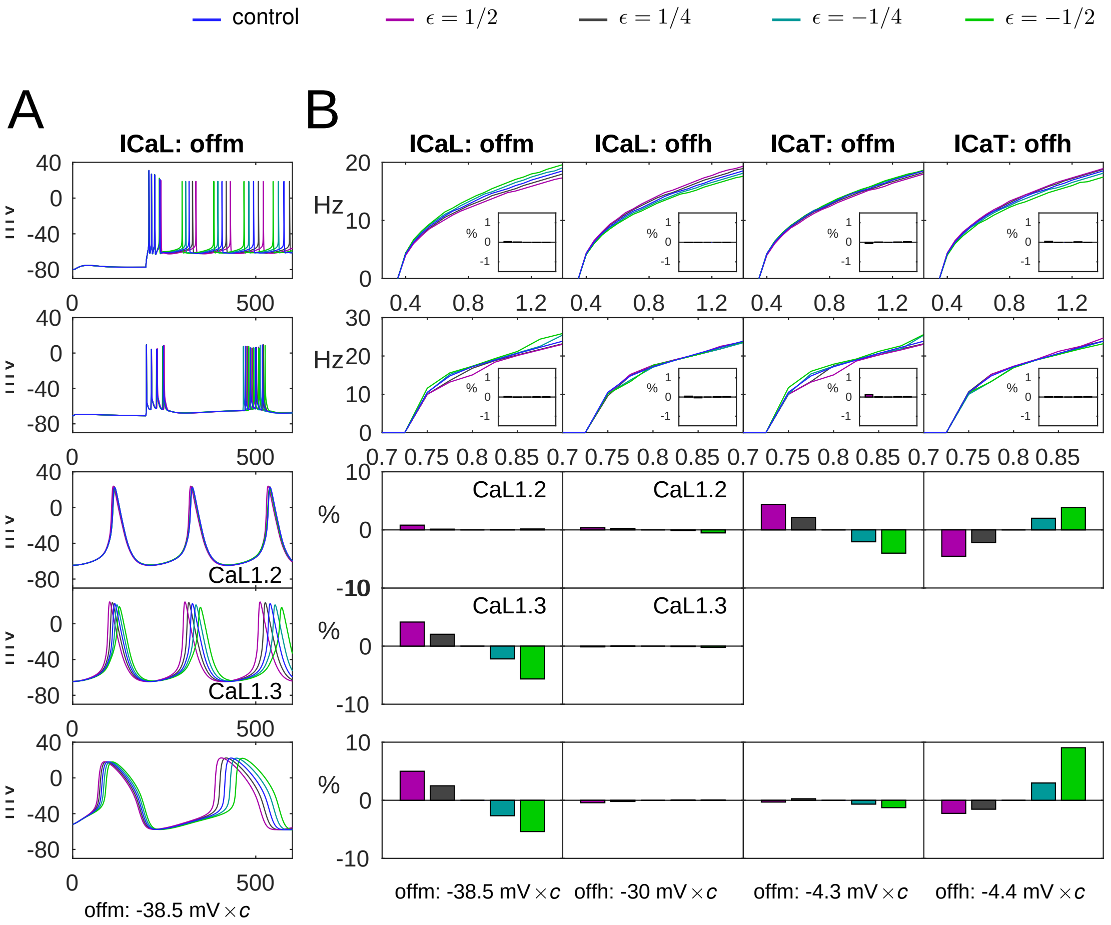


Figure S3: **Ca$^{2+}$ channel gene variants typically have opposite effects on L5PC firing and SANC pacemaking.** **A**: The membrane-potential time courses in control cells and cells implemented with a *CACNA1C* variant. See Figure 1A. **B**: The f-I curves and pacemaking rhythms of the modeled cells implemented with a Ca$^{2+}$ channel or transporter variant. See Figure 1B.

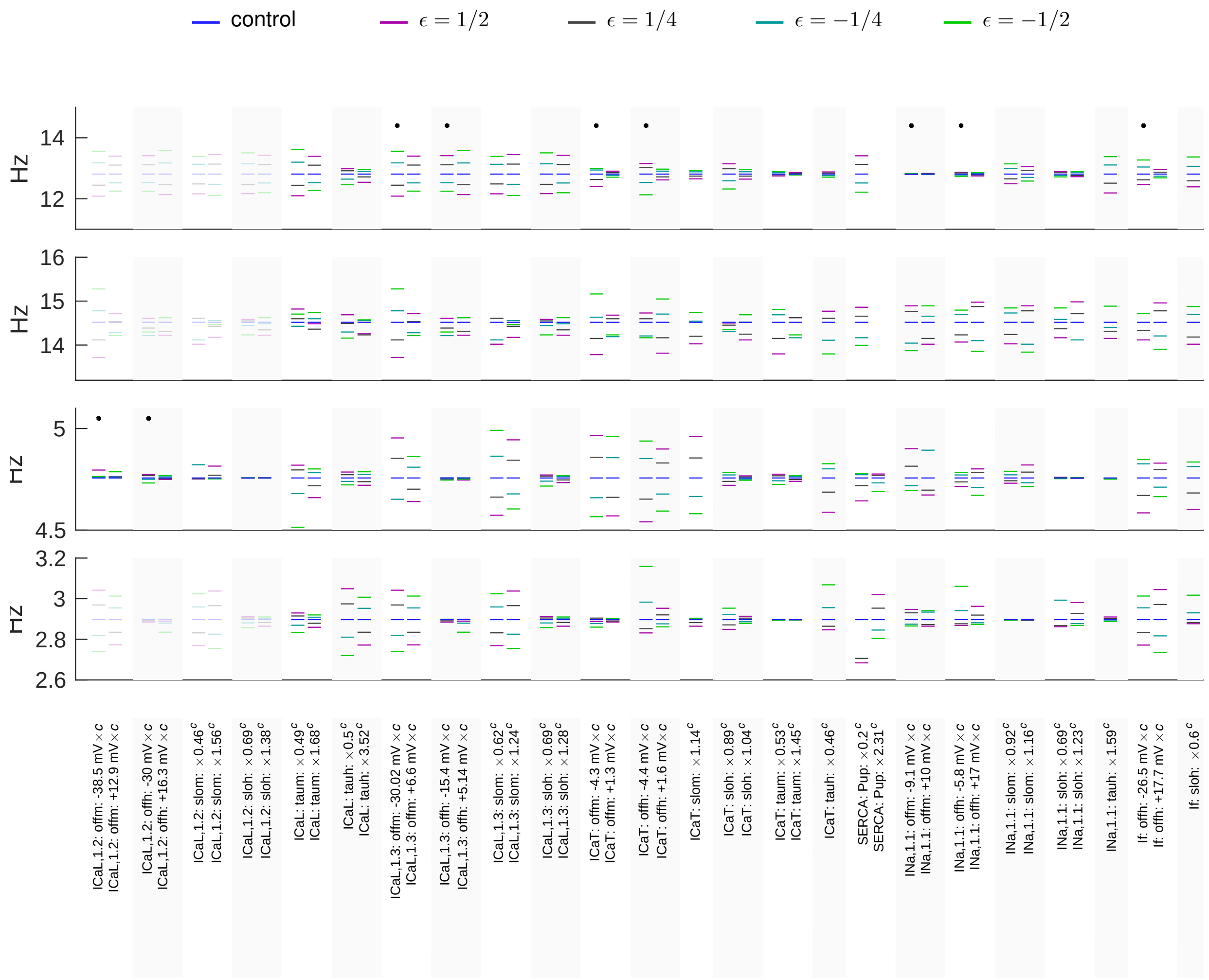


Figure S4: **Overview of single-parameter effects on L5PC firing and SANC pacemaking.** The average firing frequencies and pacemaking rhythms are shown for all artificial variants, where only a single parameter was changed. The direction and initial amplitude of the artificial variant were taken as the largest deviation from the control value across the variants of Table S1. The initial amplitude was downscaled in a similar manner as done for variants of Table S2. The left-most eight single-parameter variants are unique to the Kharche model, the data for other models is duplicated from the data of "CaL,1.3"-variants. Top panel: Firing rates in the Hay model, averaged over stimulus amplitudes 0.35–1.4 nA. Second panel: Firing rates in the Almog model, averaged over stimulus amplitudes 0.7–0.9 nA. Third panel: Pacemaking rates in the Kharche model. Bottom panel: Pacemaking rates in the Severi model. See Figures S2 and S3 for more detailed data. Variants shown in Figures S2 and S3 are highlighted with asterisks.

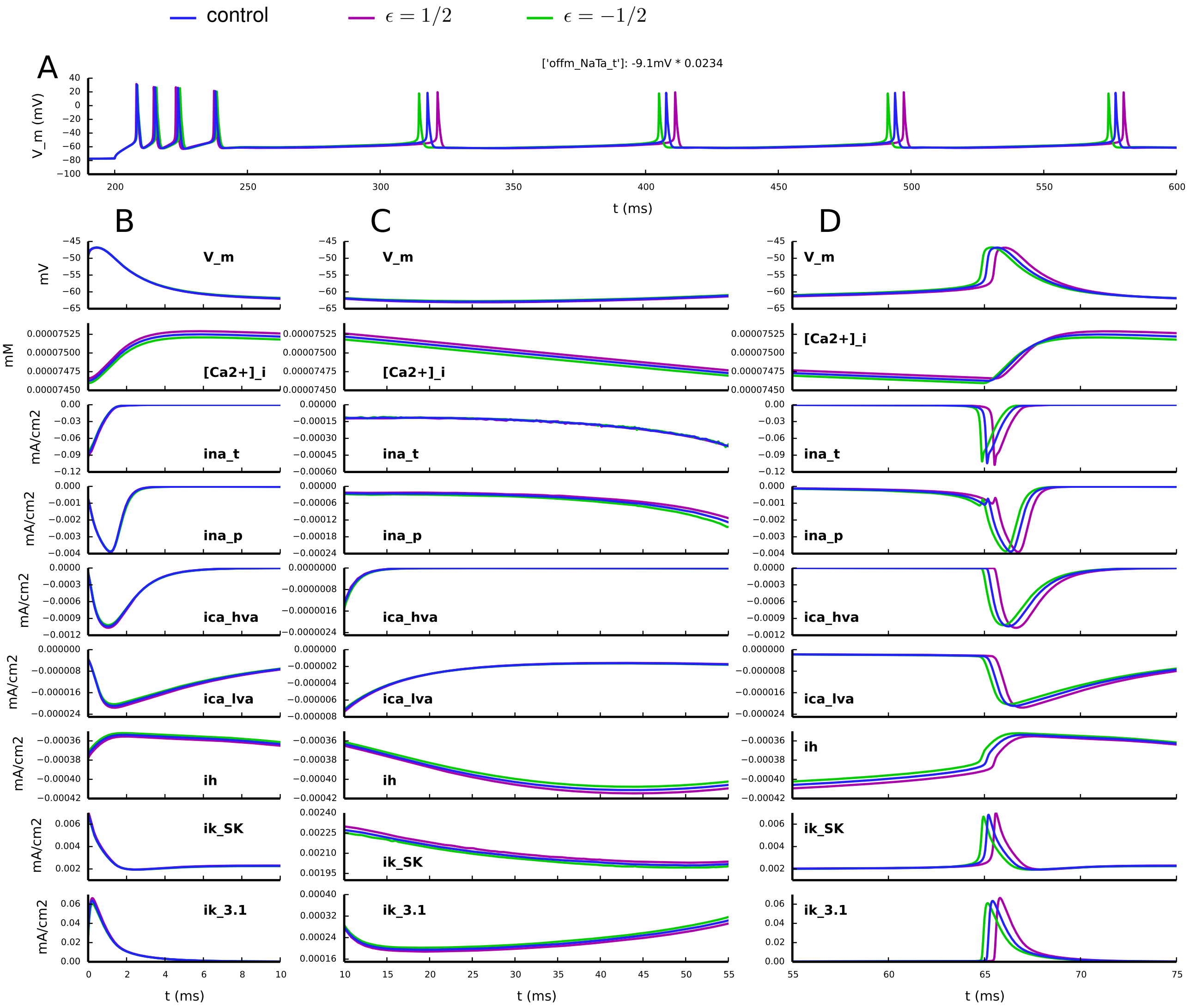


Figure S5: **Effects of lowered threshold of transient $Na^+$ currents on different current species in the Hay-model neuron in steady-state firing**. The neuron is given a somatic DC of amplitude 1.0 nA starting at 200 ms. The panels show the neuron response in control conditions (blue) and downscaled ($\epsilon = \frac{1}{2}$, magenta) variant and its opposite ($\epsilon = -\frac{1}{2}$, green). **A**: The somatic membrane potential during the first 400 ms after the DC onset. **B-D**: Time courses of the membrane potential (first row), intracellular $Ca^{2+}$ concentration (second row), and seven different current species (third to ninth row). All quantities are averaged over the whole membrane, including the membrane potential, which is why the spike amplitudes are much smaller than in panel A. The time courses of the variants are aligned such that the x axis shows the time since a somatic spike occurring near the end of the 4-second simulation — this ensures that the neuron has reached a steady firing pattern. The panel (B) shows the first 10 ms after the chosen spike, while panel (C) shows most of the inter-spike interval (10 – 55 ms) and panel (D) shows the time around the next spike (55-75 ms).

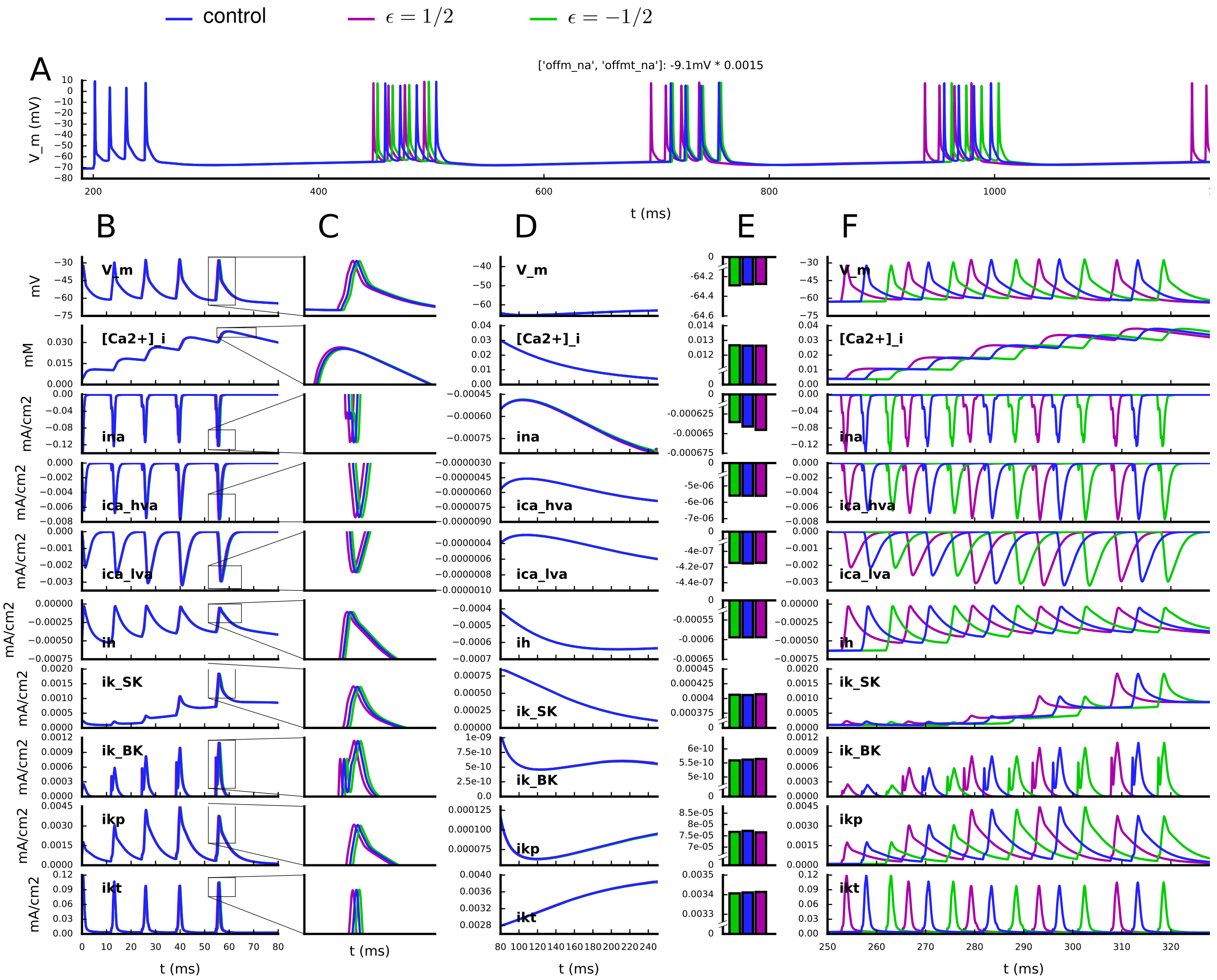


Figure S6: **Effects of lowered threshold of transient $Na^+$ currents on different current species in the Almog-model neuron in steady-state bursting**. The neuron is given a somatic DC of amplitude 0.835 nA starting at 200 ms. The panels show the neuron response in control conditions (blue) and downscaled ($\epsilon = \frac{1}{2}$, magenta) variant and its opposite ($\epsilon = -\frac{1}{2}$, green). **A**: The somatic membrane potential during the first 1000 ms after the DC onset. **B,D,F**: Time courses of the membrane potential (first row), intracellular $Ca^{2+}$ concentration (second row), and seven different current species (third to tenth row). All quantities are averaged over the whole membrane. The time courses of the variants are aligned such that the x axis shows the time since a spike occurring after a number ($n_{\mathrm{bursts}} > 3$) of initial bursts — this ensures that the neuron has reached a steady firing pattern. Within each simulation, there was variation in the inter-burst intervals, which could affect the amplitudes of the considered currents, and thus, we selected the inter-burst interval ($n_{\mathrm{bursts}}$) that most closely corresponded to the mean firing frequency of the considered variant (see Figure S2B). The panel (B) shows the time around the burst to which the chosen spike belongs (first 80 ms), while panel (D) shows most of the inter-burst interval (80 – 250 ms) and panel (F) shows the time around the next burst (250-330 ms). **C**: Zoomed-in view of the last spike in (B). **E**: Time-averaged mean of the signal in (D).

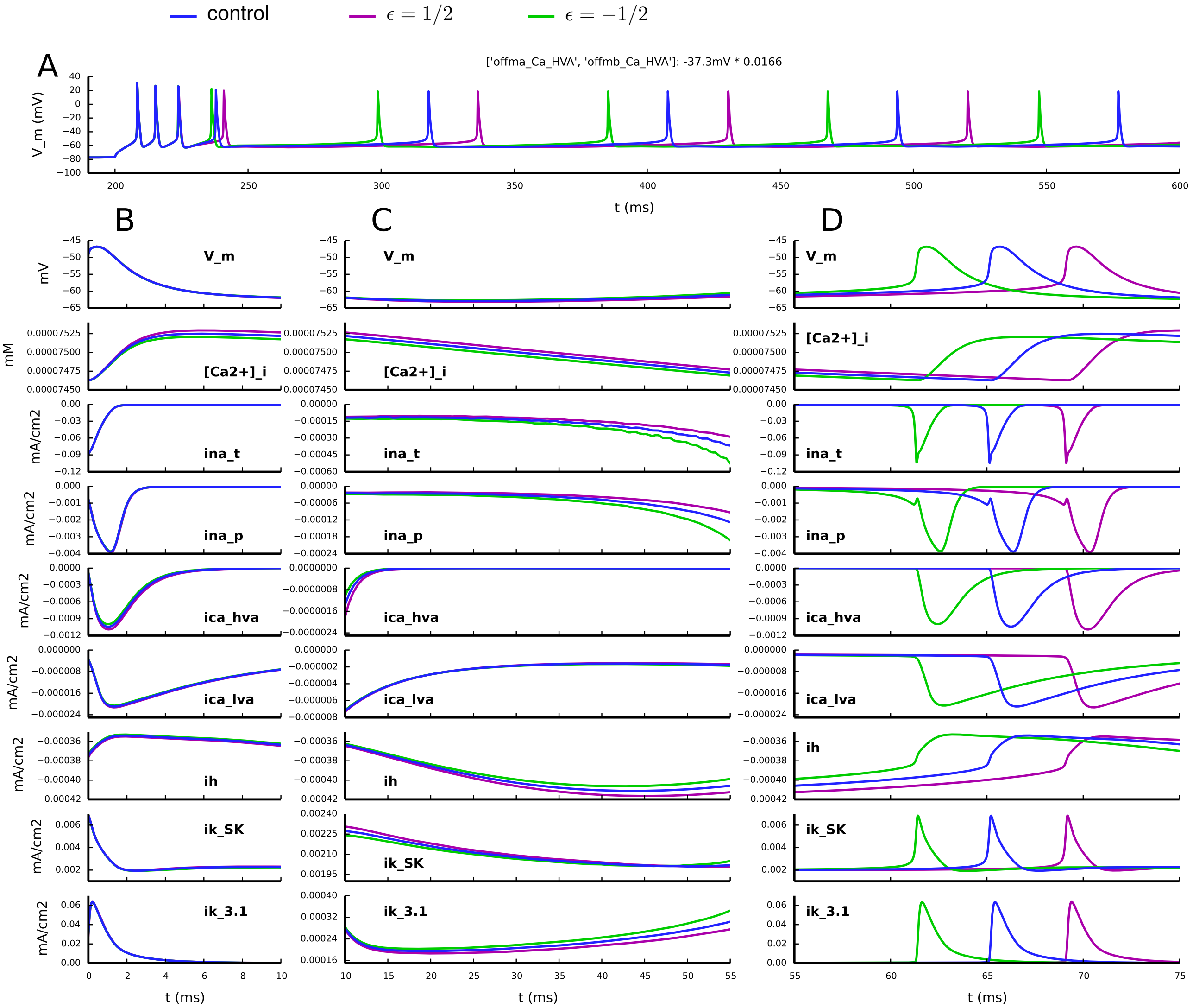


Figure S7: **Effects of lowered threshold of high-voltage-activated $Ca^{2+}$ currents on different current species in the Hay-model neuron in steady-state firing**. The neuron is given a somatic DC of amplitude 1.0 nA starting at 200 ms. The panels show the neuron response in control conditions (blue) and downscaled ($\epsilon = \frac{1}{2}$, magenta) variant and its opposite ($\epsilon = -\frac{1}{2}$, green). See Figure S5 for details.

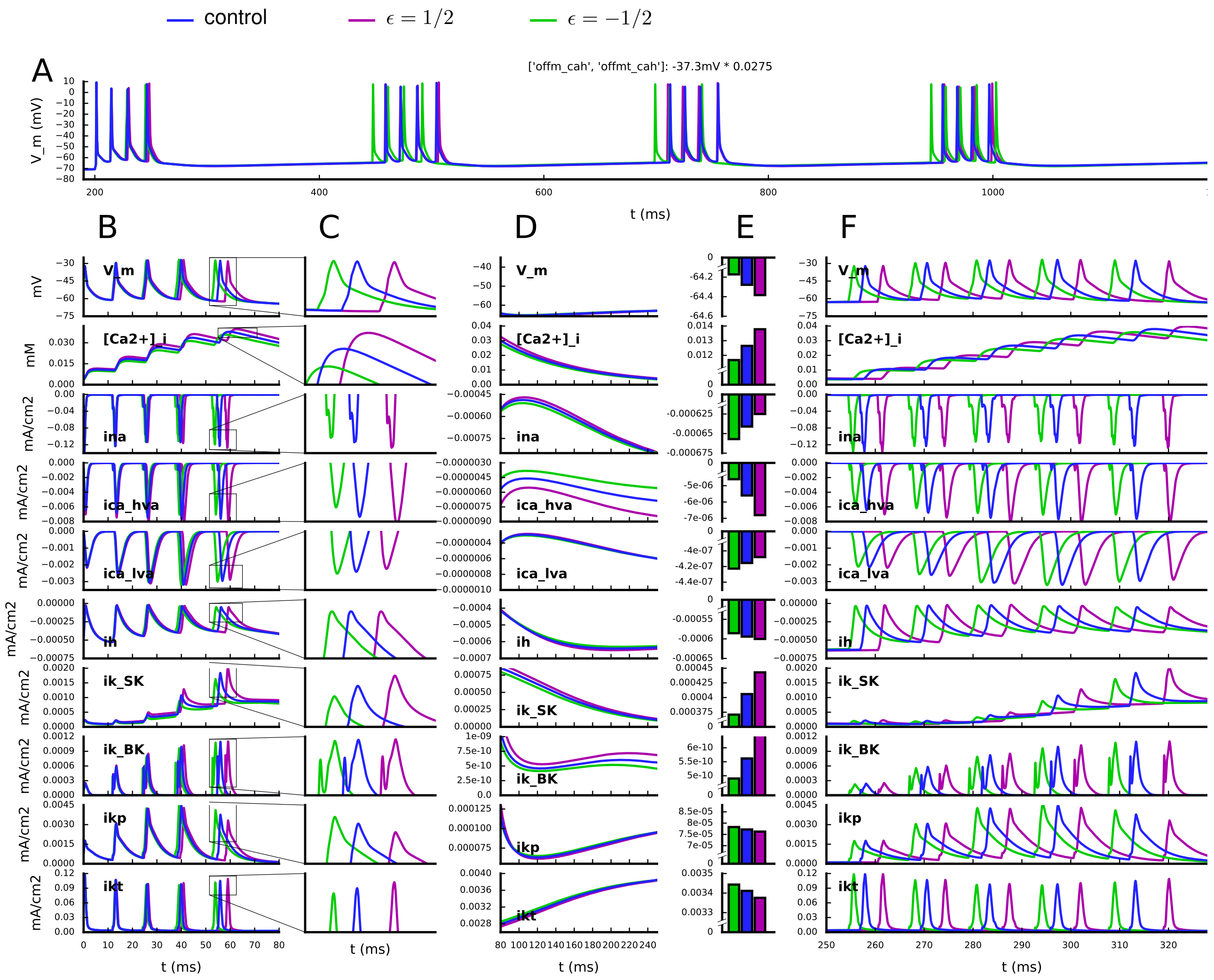


Figure S8: **Effects of lowered threshold of transient $Na^+$ currents on different current species in the Almog-model neuron in steady-state bursting**. The neuron is given a somatic DC of amplitude 0.835 nA starting at 200 ms. The panels show the neuron response in control conditions (blue) and downscaled ($\epsilon = \frac{1}{2}$, magenta) variant and its opposite ($\epsilon = -\frac{1}{2}$, green). See Figure S6 for details.

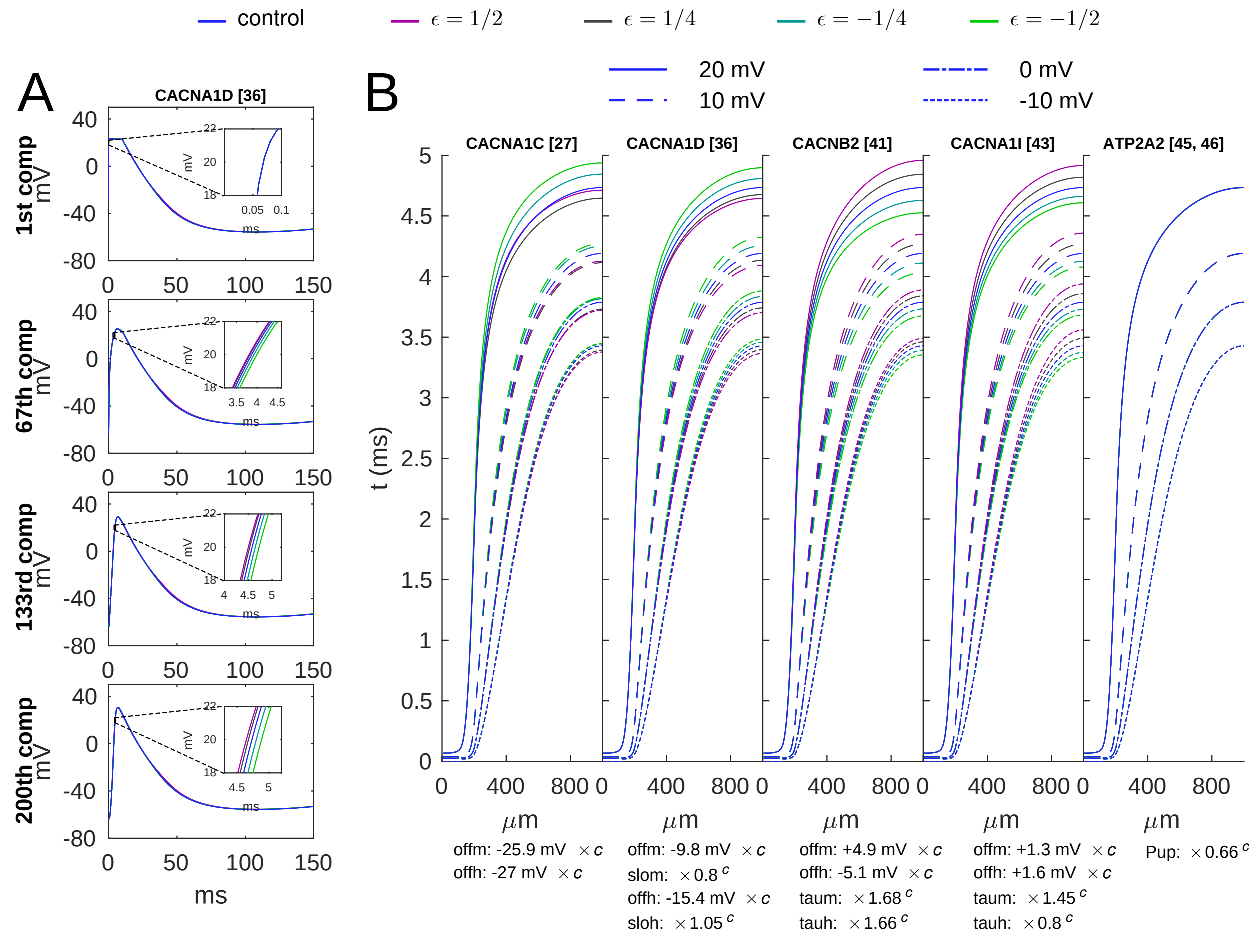


Figure S9: **Conduction velocity is changed in the Kharche model SANCs implemented with $Ca^{2+}$ channel variants.** **A**: The membrane-potential time courses in control cells and cells implemented with a *CACNA1C* variant. The membrane potentials are shown at the 1st (0 mm), 67th (0.33 mm), 133rd (0.67 mm) and last (1 mm) compartment, starting at the end of the 1000 ms hyperpolarizing pulse. The insets show a zoomed-in view around the first crossing of 20 mV membrane potential. In the three lowest panels, one can observe that the $\epsilon = \frac{1}{2}$ variant (magenta) reaches the membrane potential of 20 mV earlier than the control SANC, indicating a larger conduction velocity. **B**: The conduction delay was quantified for the variants of Figure 2. The x axis shows the distance of the compartment from the stimulated end, and y axis shows the time of first crossing of -10 mV (lowest curves, finely dashed), 0 mV (second from bottom, variably dashed), +10 mV (second from top, scarcely dashed), or +20 mV (top curves, solid). The conduction delay was high in those variants that displayed low pacemaking frequency in Figure 2, and vice versa; the *ATP2A2* variant had minuscule effects on both quantities.

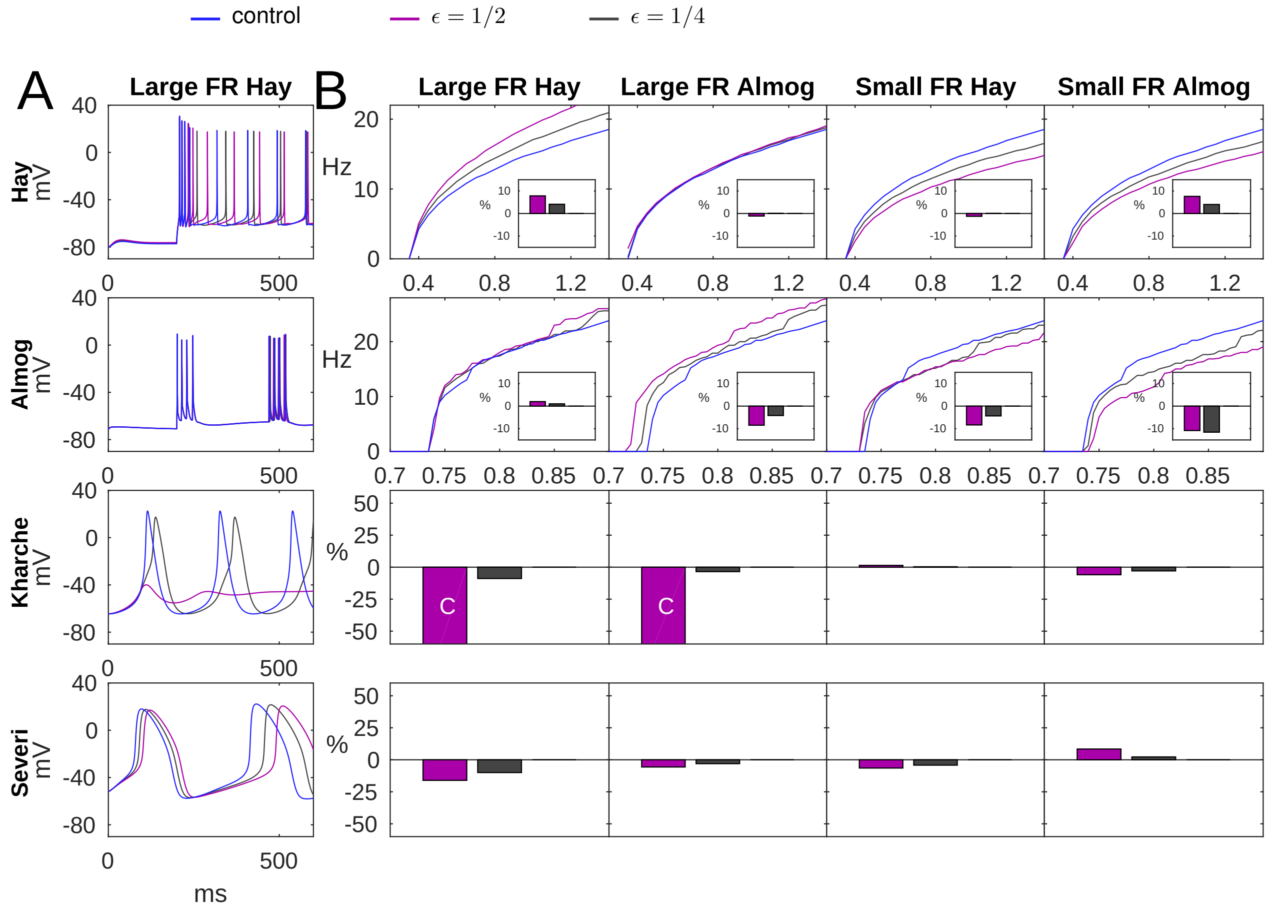


Figure S10: **Effects of combined gene variants on L5PC and SANC excitability.** **A**: The membrane-potential time courses in control cells and cells implemented with the combination of variants that increased the firing rate in a Hay-model neuron. From each gene, namely *CACNA1C*, *CACNA1D*, *CACNB2*, *CACNA1I*, *ATP2A2*, *SCN1A*, and *HCN1* the $\epsilon = \frac{1}{2}$ variant that produced the maximal firing rate in the Hay model simulations of Figure 3 was chosen. These variants were cumulatively implemented in all four models (from top to bottom: Hay, Almog, Kharche, and Severi models). Different colors represent different scalings of the variants (blue: control, magenta: combination of $\epsilon = \frac{1}{2}$ variants, gray: combination of $\epsilon = \frac{1}{4}$ variants). **B**: The f-I curves and pacemaking rhythms of the modeled cells implemented with different combinations of variants. The first column corresponds to the variant combination of (A). For the combination of the second column, the variant that produced the greatest firing rate in the Almog model was selected in a similar manner for each gene. Similar procedures were performed in the two right-most columns to choose variants that produced smallest firing rates in Hay (third column) or Almog (fourth column) model. For these combinations, genes *CACNA1C*, *CACNA1D*, *CACNB2*, *CACNA1I*, *SCN1A*, and *HCN1* were considered as the only available variant for the missing gene, *ATP2A2*, caused an increase in the firing rate in both models. For L5PCs (two upper rows), the firing rate curves are shown, whereas for SANCs, the relative difference from control cell pacemaking rhythm (4.76 Hz in the Kharche model, 2.90 Hz in the Severi model) are shown. The letter “C” in the SANC model data means that the variant made the cell cease pacemaking. The insets of the Hay and Almog model data show the change in threshold current at rest in relation to that of the control neuron (0.350 nA in the Hay model, 0.418 nA in the Almog model).

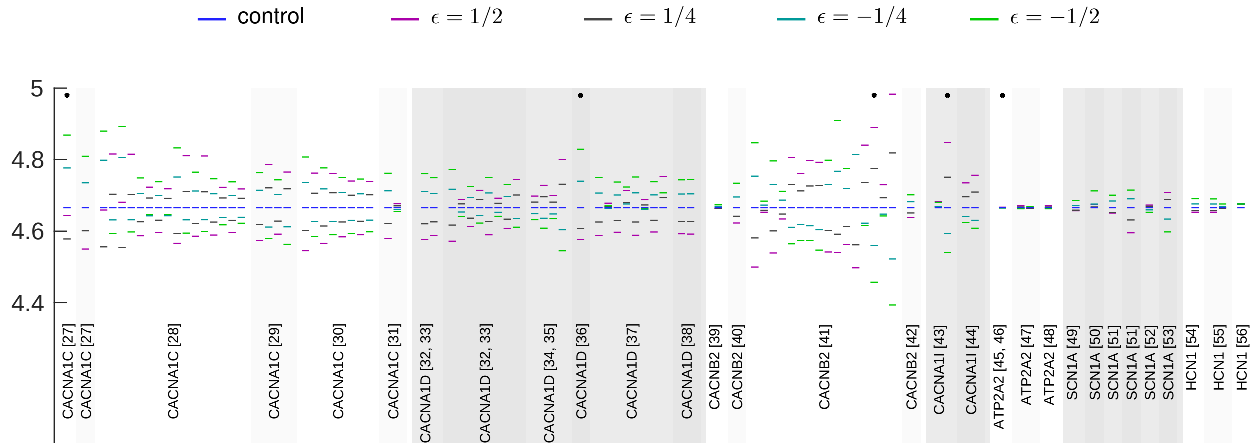


Figure S11: **Overview of variant effects on signal propagation rate in the Kharche-model cells.** The x axis shows the variant (ordered as in Table S2), and the y axis shows the time at which the last SANC reaches the +20 mV threshold since the begininning of the depolarizing voltage clamp. See Figure S9 for details. The variants of Figure S9 are marked with asterisks. The different colors represent control SANC (blue) and SANCs with downscaled variants (magenta: $\epsilon = \frac{1}{2}$, gray: $\epsilon = \frac{1}{4}$, cyan: $\epsilon = -\frac{1}{4}$, green: $\epsilon = -\frac{1}{2}$).

# References


S1. Etay Hay, Sean Hill, Felix Schürmann, Henry Markram, and Idan Segev. Models of neocortical layer 5b pyramidal cells capturing a wide range of dendritic and perisomatic active properties. *PLoS Computational Biology*, 7:e1002107, 2011.

S2. Mara Almog and Alon Korngreen. A quantitative description of dendritic conductances and its application to dendritic excitation in layer 5 pyramidal neurons. *Journal of Neuroscience*, 34(1):182–196, 2014.

S3. Elodie Christophe, Nathalie Doerflinger, Daniel J Lavery, Zoltán Molnár, Serge Charpak, and Etienne Audinat. Two populations of layer v pyramidal cells of the mouse neocortex: development and sensitivity to anesthetics. *Journal of neurophysiology*, 94(5):3357–3367, 2005.

S4. Mara Almog, Alon Korngreen, et al. Characterization of voltage-gated ca (2+) conductances in layer 5 neocortical pyramidal neurons from rats. *PLoS ONE*, 4(4):e4841–e4841, 2009.

S5. Walther Akemann, Yong-Mei Zhong, Noritaka Ichinohe, Kathleen S Rockland, and Thomas Knöpfel. Transgenic mice expressing a fluorescent in vivo label in a distinct subpopulation of neocortical layer 5 pyramidal cells. *Journal of Comparative Neurology*, 480(1):72–88, 2004.

S6. D Guan, T Tkatch, DJ Surmeier, WE Armstrong, and RC Foehring. Kv2 subunits underlie slowly inactivating potassium current in rat neocortical pyramidal neurons. *Journal of Physiology*, 581(3):941–960, 2007.

S7. Arne Battefeld, Baouyen T Tran, Jason Gavrilis, Edward C Cooper, and Maarten HP Kole. Heteromeric kv7. 2/7.3 channels differentially regulate action potential initiation and conduction in neocortical myelinated axons. *The Journal of Neuroscience*, 34(10):3719–3732, 2014.

S8. Cuiping Tian, Kaiyan Wang, Wei Ke, Hui Guo, and Yousheng Shu. Molecular identity of axonal sodium channels in human cortical pyramidal cells. *Frontiers in cellular neuroscience*, 8:297, 2014.

S9. William RJ Whitaker, Jeffrey J Clare, Andrew J Powell, Yu Hua Chen, Richard LM Faull, and Piers C Emson. Distribution of voltage-gated sodium channel $\alpha$-subunit and $\beta$-subunit mrnas in human hippocampal formation, cortex, and cerebellum. *Journal of Comparative Neurology*, 422(1):123–139, 2000.

S10. William A Catterall, Edward Perez-Reyes, Terrance P Snutch, and Joerg Striessnig. International union of pharmacology. xlviii. nomenclature and structure-function relationships of voltage-gated calcium channels. *Pharmacological reviews*, 57(4):411–425, 2005.

S11. Jenny Y Ma, William A Catterall, and Todd Scheuer. Persistent sodium currents through brain sodium channels induced by g protein $\beta\gamma$ subunits. *Neuron*, 19(2):443–452, 1997.

S12. Carmen Ballesteros-Merino, Masahiko Watanabe, Ryuichi Shigemoto, Yugo Fukazawa, John P Adelman, and Rafael Luján. Differential subcellular localization of sk3-containing channels in the hippocampus. *European Journal of Neuroscience*, 39(6):883–892, 2014.

S13. Narimane Benhassine and Thomas Berger. Homogeneous distribution of large-conductance calcium-dependent potassium channels on soma and apical dendrite of rat neocortical layer 5 pyramidal neurons. *European Journal of Neuroscience*, 21(4):914–926, 2005.

S14. Fawzia Baba-Aissa, Luc Raeymaekers, Frank Wuytack, Leonard Dode, and Rik Casteels. Distribution and isoform diversity of the organellar ca2+ pumps in the brain. *Molecular and chemical neuropathology*, 33(3):199–208, 1998.

S15. William L Stahl, Thomas J Eakin, James WM Owens, John F Breininger, Peter E Filuk, and William R Anderson. Plasma membrane ca 2+-atpase isoforms: distribution of mrnas in rat brain by in situ hybridization. *Molecular brain research*, 16(3):223–231, 1992.

S16. William L Stahl, Timothy P Keeton, and Thomas J Eakin. The plasma membrane ca 2+-atpase mrna isoform pmca 4 is expressed at high levels in neurons of rat piriform cortex and neocortex. *Neuroscience letters*, 178(2):267–270, 1994.

S17. Sanjay Kharche, Jian Yu, Ming Lei, and Henggui Zhang. A mathematical model of action potentials of mouse sinoatrial node cells with molecular bases. *American Journal of Physiology-Heart and Circulatory Physiology*, 301(3):H945–H963, 2011.

S18. Stefano Severi, Matteo Fantini, Lara A Charawi, and Dario DiFrancesco. An updated computational model of rabbit sinoatrial action potential to investigate the mechanisms of heart rate modulation. *The Journal of Physiology*, 590(18):4483–4499, 2012.

S19. Ming Lei, Sandra A Jones, Jie Liu, Matthew K Lancaster, Simon S-M Fung, Halina Dobrzynski, Patrizia Camelliti, Sebastian KG Maier, Denis Noble, and Mark R Boyett. Requirement of neuronal-and cardiac-type sodium channels for murine sinoatrial node pacemaking. *The Journal of Physiology*, 559(3):835–848, 2004.

S20. Natalie J Chandler, Ian D Greener, James O Tellez, Shin Inada, Hanny Musa, Peter Molenaar, Dario DiFrancesco, Mirko Baruscotti, Renato Longhi, Robert H Anderson, et al. Molecular architecture of the human sinus node insights into the function of the cardiac pacemaker. *Circulation*, 119(12):1562–1575, 2009.

S21. Ming Lei, Henggui Zhang, Andrew A Grace, and Christopher L-H Huang. Scn5a and sinoatrial node pacemaker function. *Cardiovascular research*, 74(3):356–365, 2007.

S22. Michael J Berridge, Peter Lipp, and Martin D Bootman. The versatility and universality of calcium signalling. *Nature Reviews Molecular Cell Biology*, 1(1):11–21, 2000.

S23. Barbara Rosati and David McKinnon. Regulation of ion channel expression. *Circulation Research*, 94(7):874–883, 2004.

S24. Michaela Kudrnac, Stanislav Beyl, Annette Hohaus, Anna Stary, Thomas Peterbauer, Eugen Timin, and Steffen Hering. Coupled and independent contributions of residues in IS6 and IIS6 to activation gating of CaV1.2. *Journal of Biological Chemistry*, 284(18):12276–12284, 2009.

S25. T. Mäki-Marttunen, G. Halnes, A. Devor, A. Witoelar, F. Bettella, S. Djurovic, Y. Wang, G. Einevoll, O. A. Andreassen, and A. M. Dale. Functional effects of schizophrenia-linked genetic variants on intrinsic single-neuron excitability: A modeling study. *Biological Psychiatry: Cognitive Neuroscience and Neuroimaging*, 1(1):49–59, 2016.

S26. Katrin Depil, Stanislav Beyl, Anna Stary-Weinzinger, Annette Hohaus, Eugen Timin, and Steffen Hering. Timothy mutation disrupts the link between activation and inactivation in cav1.2 protein. *Journal of Biological Chemistry*, 286(36):31557–31564, 2011.

S27. Annette Hohaus, Stanislav Beyl, Michaela Kudrnac, Stanislav Berjukow, Eugen N Timin, Rainer Marksteiner, Marion A Maw, and Steffen Hering. Structural determinants of l-type channel activation in segment iis6 revealed by a retinal disorder. *Journal of Biological Chemistry*, 280(46):38471–38477, 2005.

S28. Anna Stary, Michaela Kudrnac, Stanislav Beyl, Annette Hohaus, Eugen Timin, Peter Wolschann, H Robert Guy, and Steffen Hering. Molecular dynamics and mutational analysis of a channelopathy mutation in the iis6 helix of cav1.2. *Channels*, 2(3):216–223, 2008.

S29. Zhen Zhi Tang, Mui Cheng Liang, Songqing Lu, Dejie Yu, Chye Yun Yu, David T Yue, and Tuck Wah Soong. Transcript scanning reveals novel and extensive splice variations in human l-type voltage-gated calcium channel, cav1.2 $\alpha1$ subunit. *Journal of Biological Chemistry*, 279(43):44335–44343, 2004.

S30. Bao Zhen Tan, Fengli Jiang, Ming Yeong Tan, Dejie Yu, Hua Huang, Yiru Shen, and Tuck Wah Soong. Functional characterization of alternative splicing in the c terminus of l-type cav1. 3 channels. *Journal of Biological Chemistry*, 286(49):42725–42735, 2011.

S31. Gabriella Bock, Mathias Gebhart, Anja Scharinger, Wanchana Jangsangthong, Perrine Busquet, Chiara Poggiani, Simone Sartori, Matteo E Mangoni, Martina J Sinnegger-Brauns, Stefan Herzig, et al. Functional properties of a newly identified c-terminal splice variant of cav1.3 l-type ca2+ channels. *Journal of Biological Chemistry*, 286(49):42736–42748, 2011.

S32. Q. Zhang, V. Timofeyev, H. Qiu, L. Lu, N. Li, A. Singapuri, C. L. Torado, H. S. Shin, and N. Chiamvimonvat. Expression and roles of Cav1.3 ($\alpha$1D) L-type Ca2+ channel in atrioventricular node automaticity. *Journal of Molecular and Cellular Cardiology*, 50(1):194–202, 2011.

S33. Alberto Pérez-Alvarez, Alicia Hernández-Vivanco, Jose Carlos Caba-González, and Almudena Albillos. Different roles attributed to cav1 channel subtypes in spontaneous action potential firing and fine tuning of exocytosis in mouse chromaffin cells. *Journal of Neurochemistry*, 116(1):105–121, 2011.

S34. Alexandra Pinggera, Andreas Lieb, Bruno Benedetti, Michaela Lampert, Stefania Monteleone, Klaus R Liedl, Petronel Tuluc, and Jörg Striessnig. Cacna1d de novo mutations in autism spectrum disorders activate cav1.3 l-type calcium channels. *Biological Psychiatry*, 77(9):816–822, 2015.

S35. Elena AB Azizan, Hanne Poulsen, Petronel Tuluc, Junhua Zhou, Michael V Clausen, Andreas Lieb, Carmela Maniero, Sumedha Garg, Elena G Bochukova, Wanfeng Zhao, et al. Somatic mutations in atp1a1 and cacna1d underlie a common subtype of adrenal hypertension. *Nature Genetics*, 45(9):1055–1060, 2013.

S36. Andreas Lieb, Anja Scharinger, Simone Sartori, Martina J Sinnegger-Brauns, and Jörg Striessnig. Structural determinants of cav1. 3 l-type calcium channel gating. *Channels*, 6(3):197–205, 2012.

S37. Jonathan M Cordeiro, Mark Marieb, Ryan Pfeiffer, Kirstine Calloe, Elena Burashnikov, and Charles Antzelevitch. Accelerated inactivation of the L-type calcium current due to a mutation in CACNB2b underlies brugada syndrome. *Journal of Molecular and Cellular Cardiology*, 46(5):695–703, 2009.

S38. Enrique Massa, Kevin M Kelly, David I Yule, Robert L MacDonald, and Michael D Uhler. Comparison of fura-2 imaging and electrophysiological analysis of murine calcium channel $\alpha$ 1 subunits coexpressed with novel $\beta$ 2 subunit isoforms. *Molecular Pharmacology*, 47(4):707–716, 1995.

S39. Sabine Link, Marcel Meissner, Brigitte Held, Andreas Beck, Petra Weissgerber, Marc Freichel, and Veit Flockerzi. Diversity and developmental expression of L-type calcium channel $\beta$2 proteins and their influence on calcium current in murine heart. *Journal of Biological Chemistry*, 284(44):30129–30137, 2009.

S40. Dan Hu, Hector Barajas-Martinez, Vladislav V Nesterenko, Ryan Pfeiffer, Alejandra Guerchicoff, Jonathan M Cordeiro, Anne B Curtis, Guido D Pollevick, Yuesheng Wu, Elena Burashnikov, and Charles Antzelevitch. Dual variation in scn5a and cacnb2b underlies the development of cardiac conduction disease without brugada syndrome. *Pacing and Clinical Electrophysiology*, 33(3):274–285, 2010.

S41. Janet Murbartián, Juan Manuel Arias, and Edward Perez-Reyes. Functional impact of alternative splicing of human T-type Cav3.3 calcium channels. *Journal of Neurophysiology*, 92(6):3399–3407, 2004.

S42. Juan Carlos Gomora, Janet Murbartian, Juan Manuel Arias, Jung-Ha Lee, and Edward Perez-Reyes. Cloning and expression of the human t-type channel cav3.3: insights into prepulse facilitation. *Biophysical Journal*, 83(1):229–241, 2002.

S43. Muthu Periasamy, Thomas D Reed, Lynne H Liu, Yong Ji, Evgeny Loukianov, Richard J Paul, Michelle L Nieman, Tara Riddle, John J Duffy, Thomas Doetschman, et al. Impaired cardiac performance in heterozygous mice with a null mutation in the sarco (endo) plasmic reticulum ca2+-atpase isoform 2 (serca2) gene. *Journal of Biological Chemistry*, 274(4):2556–2562, 1999.

S44. Yong Ji, M Jane Lalli, Gopal J Babu, Yanfang Xu, Darryl L Kirkpatrick, Lynne H Liu, Nipavan Chiamvimonvat, Richard A Walsh, Gary E Shull, and Muthu Periasamy. Disruption of a single copy of the serca2 gene results in altered ca2+ homeostasis and cardiomyocyte function. *Journal of Biological Chemistry*, 275(48):38073–38080, 2000.

S45. Leonard Dode, Jens Peter Andersen, Natalie Leslie, Jittima Dhitavat, Bente Vilsen, and Alain Hovnanian. Dissection of the functional differences between sarco (endo) plasmic reticulum ca2+-atpase (serca) 1 and 2 isoforms and characterization of darier disease (serca2) mutants by steady-state and transient kinetic analyses. *Journal of Biological Chemistry*, 278(48):47877–47889, 2003.

S46. Wooin Ahn, Min Goo Lee, Kyung Hwan Kim, and Shmuel Muallem. Multiple effects of serca2b mutations associated with darier's disease. *Journal of Biological Chemistry*, 278(23):20795–20801, 2003.

S47. Sandrine Cestèle, Paolo Scalmani, Raffaella Rusconi, Benedetta Terragni, Silvana Franceschetti, and Massimo Mantegazza. Self-limited hyperexcitability: Functional effect of a familial hemiplegic migraine mutation of the Nav1.1 (SCN1A) na+ channel. *Journal of Neuroscience*, 28(29):7273–7283, 2008.

S48. Kaate RJ Vanmolkot, Elena Babini, Boukje de Vries, Anine H Stam, Tobias Freilinger, Gisela M Terwindt, Lisa Norris, Joost Haan, Rune R Frants, Nabih M Ramadan, et al. The novel p.L1649Q mutation in the SCN1A epilepsy gene is associated with familial hemiplegic migraine: Genetic and functional studies. *Human Mutation*, 28(5):522–522, 2007.

S49. Linda Volkers, Kristopher M Kahlig, Nienke E Verbeek, Joost HG Das, Marjan JA van Kempen, Hans Stroink, Paul Augustijn, Onno van Nieuwenhuizen, Dick Lindhout, Alfred L George, et al. Nav1.1 dysfunction in genetic epilepsy with febrile seizures-plus or dravet syndrome. *European Journal of Neuroscience*, 34(8):1268–1275, 2011.

S50. Sandrine Cestèle, Angelo Labate, Raffaella Rusconi, Patrizia Tarantino, Laura Mumoli, Silvana Franceschetti, Grazia Annesi, Massimo Mantegazza, and Antonio Gambardella. Divergent effects of the t1174s scn1a mutation associated with seizures and hemiplegic migraine. *Epilepsia*, 54(5):927–935, 2013.

S51. Massimo Mantegazza, Antonio Gambardella, Raffaella Rusconi, Emanuele Schiavon, Ferdinanda Annesi, Rita Restano Cassulini, Angelo Labate, Sara Carrideo, Rosanna Chifari, Maria Paola Canevini, et al. Identification of an nav1.1 sodium channel (scn1a) loss-of-function mutation associated with familial simple febrile seizures. *Proceedings of the National Academy of Sciences*, 102(50):18177–18182, 2005.

S52. Takahiro M Ishii, Noriyuki Nakashima, and Harunori Ohmori. Tryptophan-scanning mutagenesis in the s1 domain of mammalian hcn channel reveals residues critical for voltage-gated activation. *Journal of Physiology*, 579(2):291–301, 2007.

S53. Heinte Lesso and Ronald A Li. Helical secondary structure of the external s3-s4 linker of pacemaker (hcn) channels revealed by site-dependent perturbations of activation phenotype. *Journal of Biological Chemistry*, 278(25):22290–22297, 2003.

S54. Konstantin Wemhöner, Tatyana Kanyshkova, Nicole Silbernagel, Juncal Fernandez-Orth, Stefan Bittner, Aytug K Kiper, Susanne Rinné, Michael F Netter, Sven G Meuth, Thomas Budde, et al. An n-terminal deletion variant of hcn1 in the epileptic wag/rij strain modulates hcn current densities. *Frontiers in Molecular Neuroscience*, 8:63, 2015.

S55. Olga Fedorenko, Nathalie Strutz-Seebohm, Ulrike Henrion, Oana N Ureche, Florian Lang, Guiscard Seebohm, and Undine E Lang. A schizophrenia-linked mutation in pip5k2a fails to activate neuronal m channels. *Psychopharmacology*, 199(1):47–54, 2008.

S56. Mulugu V Brahmajothi, Michael J Morales, Donald L Campbell, Charles Steenbergen, and Harold C Strauss. Expression and distribution of voltage-gated ion channels in ferret sinoatrial node. *Physiological Genomics*, 42(2):131–140, 2010.

S57. Arie O Verkerk, Ronald Wilders, MM Van Borren, Hanno L Tan, et al. Is sodium current present in human sinoatrial node cells. *Int J Biol Sci*, 5(2):201–204, 2009.

S58. S Hong Lee, Teresa R DeCandia, Stephan Ripke, Jian Yang, Patrick F Sullivan, Michael E Goddard, Matthew C Keller, Peter M Visscher, Naomi R Wray, Schizophrenia Psychiatric Genome-Wide Association Study Consortium, et al. Estimating the proportion of variation in susceptibility to schizophrenia captured by common snps. *Nature Genetics*, 44(3):247–250, 2012.

S59. Pietro Mesirca, Angelo G Torrente, and Matteo E Mangoni. Functional role of voltage gated ca2+ channels in heart automaticity. *Frontiers in Physiology*, 6:19, 2015.

S60. H Zhang, AV Holden, I Kodama, H Honjo, M Lei, T Varghese, and MR Boyett. Mathematical models of action potentials in the periphery and center of the rabbit sinoatrial node. *American Journal of Physiology-Heart and Circulatory Physiology*, 279(1):H397–H421, 2000.
